# T-pattern detection in the scientific literature of this century: A systematic review

**DOI:** 10.3389/fpsyg.2023.1085980

**Published:** 2023-03-01

**Authors:** M. Teresa Anguera, Gudberg K. Jonsson, Elena Escolano-Pérez, Carmen Rosa Sánchez-Lopez, José Luis Losada, Mariona Portell

**Affiliations:** ^1^Faculty of Psychology, Institute of Neurosciences, University of Barcelona, Barcelona, Spain; ^2^Human Behavior Laboratory, School of Health Sciences, University of Iceland, Reykjavík, Iceland; ^3^Faculty of Education, University of Zaragoza, Zaragoza, Spain; ^4^Department of Clinical Psychology, Psychobiology and Methodology, University of La Laguna, San Cristóbal de La Laguna, Spain; ^5^Faculty of Psychology, University of Barcelona, Barcelona, Spain; ^6^Department of Psychobiology and Methodology of Health Sciences, Universitat Autònoma de Barcelona, Cerdanyola del Vallès, Spain

**Keywords:** systematic review, PRISMA guidelines, T-pattern, TPA, THEME, mixed methods

## Abstract

**Introduction:**

Scientific literature contains mainly systematic reviews focused on substantial aspects, but there are also approaches that have combined both substantial and methodological aspects, which is our preferred option since it undeniably adds value. The aims of this study were: (1) to carry out a systematic review of the literatura on T-Pattern analysis (TPA), and (2) to explore the possible contribution of mixed methods research to the integration of qualitative and quantitative elements on a synthesis level.

**Methods:**

Based on PRISMA guidelines, searches were carried out in the Scopus, PsycINFO, and Web of Science databases. The general search syntax was: “THEME” AND (“T-Patterns” OR “T Patterns”) carried out in title, keywords and abstract. In addition, we included empirical articles on THEME and T-Patterns collected in other sources based on citations in several empirical works and consultations with different authors. This selection process resulted in 125 primary documents making up this systematic review.

**Results:**

The results showed that the detection of structures in behavior patterns forms a nexus between studies carried out in very diverse fields and contexts. Most studies are observational, whilst the applicability and power of T-Pattern detection are extraordinary. It allows the researcher to go deeper in a robust analysis that responds to the integration of qualitative and quantitative elements which constitutes the leit motive of mixed methods; and also to discover the deep, hidden structure that underlies the respective databases, regardless of the methodology used in each study. The possibilities in assigning parameters notably increase the options for obtaining results and their interpretation.

**Discussion:**

It is relevant the extraordinary strength and applicability of T-pattern detection. There is a high presence of T-pattern detection and analysis in studies using observational methodology. It is necessary commit to consolidating the methodological analysis of selected works, as taking individual and collective responsibility for improving methodological quality of TPA studies, taking advantage of the resources provided by the THEME program.

## 1. Introduction

### 1.1. Toward a systematic review focused on methodology

The systematic review is a special type of literature review that confers added advantages, characterized by being “methodical, comprehensive, transparent, and replicable” (Siddaway et al., 2019, p. 751), and its use in decision making has rendered it extremely effective, especially given the significant increase in scientific literature (Anderson et al., 2013). The general requirement of the systematic review is to obtain a comprehensive synthesis of evidence (Higgins and Green, 2011).

The great advantage of systematic reviews, within their plurality, is that they enable the researcher to summarize many works that have a common nexus —specified as the focus— and to organize scientific evidence (Pluye et al., 2016). The expression *systematic review* was popularized in the 1990s, and its main defining feature is that it uses explicit criteria and procedures to identify, critically assess and synthesize relevant literature. As Greenhalgh points out (Greenhalgh, 1997, p. 672): “A systematic review is an overview of primary studies which contains an explicit statement of objectives, materials and methods and has been conducted according to explicit and reproducible methodology.”

One of the challenges of the systematic review that Hawker et al. (2002) perceived at the beginning of this century, was the inclusion of evidence from different perspectives and methodologies; and their intention was to create a database that would serve as a resource for other researchers. We too are equally interested in combining the advantages of the conventional systematic review with a methodological approach, as we have demonstrated in previous works (Sarmento et al., 2018; Preciado et al., 2019, 2021; Alarcón-Espinoza et al., 2022; Tronchoni et al., 2022), thus going deeper into methodological development.

Scientific literature contains mainly systematic reviews that have focused on substantial aspects, but there are also approaches that have combined both substantial and methodological aspects (Durach et al., 2017), which is our preferred option since we believe it undeniably adds value. As Smalborne and Quinton (2011) affirm, systematic reviews in turn create an analytical framework for analyzing primary data, and our commitment is to consolidating the methodological analysis of the selected works.

In this sense, Hong and Pluye (2019) consider that by taking methodological aspects into account, new challenges arise in relation with how to carry out a critical assessment of the selected primary documents, and which differ from the methodologies used (Harden and Thomas, 2005). In order to tackle this challenge, it is necessary to delve deeper into the understanding of primary document profiles with a view to synthesizing and integrating the evidence contained in them (Hong et al., 2017); while Hong and Pluye (2019) suggest using critical appraisal, which has been successful in systematic reviews over the last few years (Katrak et al., 2004; Bai et al., 2012).

This systematic review arises from the desire to carry out a transparent synthesis study (Smalborne and Quinton, 2011) focusing on the common nexus in methodological aspects that cuts across two points. The main point is the review of the use of T-pattern detection, exploring their application within the framework of observational methodology (Anguera, 1979, 2003; Anguera et al., 2019, 2021) in comparison to other methodological approaches. The second methodological point that singularizes this study is that it places it in the crossroads of systematic review and mixed methods. Throughout the remainder of the introduction, we will both summarize the framework derived from the interaction between the systematic review and mixed methods, and also justify the interest of this systematic review of T-patterns.

### 1.2. The systematic review from mixed methods

In previous works we have dealt with the relevance of mixed methods, specifically how observational studies —both direct (Anguera and Hernández-Mendo, 2016; Anguera et al., 2017a) and indirect observation (Anguera et al., 2017b,^2018^; Anguera, 2020)— can be considered mixed-method in themselves.

Over the last few years there has been an exponential growth in scientific literature relating to mixed methods, which is also undeniably relevant within the systematic review as well as in other types of synthesizing research evidence. Systematic reviews have traditionally shown a preference for quantitative evidence (Hong et al., 2017), but interest in qualitative evidence has grown progressively, especially in: the integrative review (Whittemore and Knafl, 2005), mixed-method review (Harden and Thomas, 2005), mixed-method research synthesis (Heyvaert et al., 2016), mixed research synthesis (Sandelowski et al., 2006), and mixed studies review (Pluye et al., 2009; Pluye and Hong, 2014). As Hong et al. (2017) reaffirm, these reviews enable a greater understanding of quantitative evidence, of qualitative evidence, and a corroboration of the knowledge obtained from both.

Quantitative output is based on the numerical values of variables or dimensions and on the results of statistical analysis, whilst it is considered qualitative when data is interpreted or summarized to generate outputs such as concepts, categories or theories. However, the distinction between qualitative and quantitative analysis is not clear, particularly since the interest in *continuum* between quantitative and qualitative poles has been gaining ground (Onwuegbuzie et al., 2011; Anguera, 2022).

In this sense, we can affirm the existence of a wide range of possibilities. Considering there are no quantitative methods that do not imply qualitative elements in some stages of the process (Chang et al., 2009; Sandelowski, 2014), nor research that is “inherently quantitative, qualitative, or mixed-method” (Newman and Hitchcock, 2011, p. 382), and “radical middle point” (Onwuegbuzie, 2012, p. 210) stands out. This represents an added value which opens the MIXED space (M: Methodological thinker; I: Integrative, integrated, and integral researcher; X: Xenophilous researcher; E: Empower; D: Development) that will mesh with the *mixed analysis crossover* (Onwuegbuzie and Johnson, 2021) where the analyses of the primary documents can be found, and which reaffirms the *continuum* between qualitative and quantitative elements rather than the opposition.

Over the last few years interesting advances have been made relating to qualitative and quantitative evidence review, centered both on quality (Pluye et al., 2009; Crowe and Sheppard, 2011; Sirriyeh et al., 2012) and on the integration of evidence (Dixon-Woods et al., 2004; Mays et al., 2005; Tricco et al., 2016), at the same time that new modalities of synthesis have been proposed (Hong et al., 2017).

Heyvaert et al. (2013) illustrate how mixed methods contribute to the integration of qualitative and quantitative research in terms of synthesis. On a primary level, the researcher collects qualitative and quantitative data from the participants (interviews, systematic observation, surveys, etc.), combining them in a study; whilst in terms of synthesis, the systematic review applies the principles of mixed-method research, coming together in *mixed methods research synthesis* (MMRS). Even though the scientific literature about mixed methods on a primary level is exponential, much less attention has been paid to the possibilities of integration on a synthesis level (Sandelowski et al., 2006; Dellinger and Leech, 2007; Voils et al., 2008); although different terms have been coined to refer to ways of synthesizing empirical evidence (Heyvaert et al., 2013); such as *systematic review*, *integrative review*, *research synthesis*, *realist synthesis*, *qualitative review*, *narrative review*, *meta-analysis.*

Prior to the implementation of the *mixed methods research synthesis* (MMRS) modality (Harden and Thomas, 2010; Heyvaert et al., 2013), historically two main approaches to synthesis studies had been developed which highlighted the systematic review as a qualitative modality, and meta-analysis as a quantitative modality.

In the last few years there has been a growing interest in synthesizing evidence derived from studies with differing designs, and with qualitative, quantitative and mixed-method approaches. Similarly, there have been methodological advances in the integration of qualitative and quantitative evidence (Hong et al., 2017), along with those relating to the quality of primary documents (Pluye et al., 2009; Crowe and Sheppard, 2011; Sirriyeh et al., 2012).

Within the framework of primary studies that form the basis of systematic reviews, we find qualitative data (observational records, interview transcripts, diverse documents, etc.), with the predictable aim of adequately interpreting the proposals of the actors involved. Nevertheless, there are essentially two main problems that may arise, depending on the level of abstraction. On the one hand there is the analysis of patterns of simultaneous occurrence or lack of co-occurrence —if the risk of disaggregation is not avoided— that would imply transforming multi-dimensionality into one-dimensionality (Sivesind, 1999), thus impoverishing the batch information by reducing the length of the event-types in THEME, which is at the core of this research.

The connection between phases plays a crucial role in integration, and has recently been ratified by Pluye et al. (2018). We propose to adopt *quantitizing*, schematized in QUAL-QUAN-QUAL (Anguera et al., 2020), as a guide for the methodological analysis of primary documents in this systematic review. This allows us to move upwards in the integration typical of mixed methods on a synthesis level, tying in assimilation and configuration on the one hand, and dimensionality and case aggregation on the other.

Given that this systematic review of T-pattern detection has been carried out from a mixed-method approach, it is worth mentioning the words of Magnusson (2020a, p. 2):

As a Mixed Methods approach, T-pattern analysis (TPA) passes repeatedly between qualitative and quantitative analyses, from data collection logging the occurrences of qualities (categories) and their real-time (quantitative) locations resulting in time-stamped data, here T-data, to the detection of T-patterns (qualities) […], typically followed by both qualitative and quantitative analyses of the detected patterns.

### 1.3. The interest of a systematic review of T-patterns

The research question undoubtedly determines the structure and reach of the systematic review, and needs to be clearly defined (Perestelo-Pérez, 2013).

Our reasons for focusing on T-pattern analysis are as follows: (a) The relevance of an analysis centered on the description and detection of complex real-time patterns, which provides unsubstitutable analytical resources to psychological research; (b) the scientific community has at its disposal the computer program THEME, which, almost in its 7th version, and an academic version freely available for number of years; and (c) its extremely high applicability in the fields of psychology (Agliati et al., 2005; Diana et al., 2018 -primary document 37-; Portell et al., 2019 -primary document 90-), sport (Lapresa et al., 2013a -primary document 69-; Castañer et al., 2016 -primary document 30-; Amatria et al., 2017 -primary document 6-), ethology (Kerepesi et al., 2006 -primary document 66-; Jonsson et al., 2010), health (Blanchet et al., 2005 -primary document 66-; Haynal-Reymond et al., 2005; Arias-Pujol and Anguera, 2020 -primary document 10-), education (Suárez et al., 2018 -primary document 111-; Terrenghi et al., 2019; Escolano-Pérez, 2020 -primary document 39-), etc., regardless of the methodology used, whether it be observational (Gutiérrez-Santiago et al., 2011 -primary document 53-; Escolano-Pérez et al., 2019 -primary document 40-; Terroba et al., 2021 -primary document 116-) or experimental (Hocking et al., 2007 -primary document 58-; Casarrubea et al., 2015), and the scale, from micro (Hirschenhauser et al., 2002; Nicol et al., 2015) to macro (Koch et al., 2005).

**FIGURE 1:**
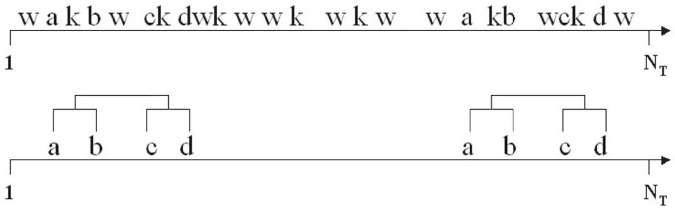
T-pattern detection (Magnusson, 2000, p. 95), with permission of the author.

The T-pattern project began in 1970 in the field of ethology (Magnusson, 1981), studying social interaction and organization in insects and primates, including humans, inspired by the work of Lorenz, von Frisch and Tinbergen. Throughout the decades since then, Magnusson (1975; 1978; 1981; 1996; 2000; 2005; 2006; 2016; 2017; 2018; 2020a,b; Magnusson et al., 2016) has worked unceasingly on the definition and mathematical development of T-patterns, or temporal patterns, as well as on the construction of the necessary algorithms. A T-Pattern is defined as the structure formed by a series of events that take place concurrently or sequentially with greater frequency than would randomly be expected if all the events were independently distributed. These events —that in observational methodology terms we shall call multi-events (Bakeman and Quera, 1996)— occur in the same order, maintaining temporal distances between them that remain invariant, or at least relatively, with respect to the null hypothesis that each event is independent and randomly distributed temporally (Magnusson, 1996, 2000). According to Magnusson (2000, pp. 94–95), when THEME detects an occurrence of “A” followed by “B” within a critical interval, it generates a simple T-pattern (AB). Occurrences of simple T-patterns become events, which are then treated as initial event-types at the subsequent detection level. Theme repeats this process, level by level (from 1 to n) in search of critical interval relationships featuring T-patterns detected in previous levels. Accordingly, all T-patterns, Q = X_1_ X_2_… X_m_, can be divided into at least two events within a critical interval. In other words QLeft [d1,d2] QRight; QLeft and QRight can be part of a more complex T-pattern X1…Xm expressed as the terminals of a binary-tree. In other words, critical interval relationships may be detected between a simple T-pattern (AB) and an event-type K, giving rise to a level-2 T-pattern with three events [(AB)K] or (see Figure 1) between two simple T-patterns (AB) and (CD), giving rise to a more complex level-2 T-pattern with four events [(AB)(CD)].

The essence of a T-pattern project is the discovery of hidden structures from the critical interval between point series with respect to the temporal dimension; thus revealing itself to be a highly valuable analytical instrument which, at the same time, entails a permanent dialogue with the respective conceptual framework.

The basic premise of T-pattern detection is that the interactive flow, or chain of behaviors, consists of structures of variable stability that can be visualized through the detection of underlying T-patterns (Suárez et al., 2018 -primary document 111-; Portell et al., 2019 -primary document 90-; Arias-Pujol and Anguera, 2020 -primary document 10-; Santoyo et al., 2020 -primary document 105-). It is not easily visible nature increases its potential for discovery, given that the researcher’s interest lies in being able to extract the internal structure that shows the key to the occurring behavior (Arbulu et al., 2016). One great advantage of T-pattern detection lies in the fact that it is not constrained by implicit suppositions about the distribution of studied behaviors; and it enables the selection of minimum number of occurrences and significance level –among other parameters– thus aiming to achieve a clear control over random discoveries.

The relevance that interest in T-pattern detection has gained, along with the applicability it has shown in the last few years justify this systematic review, whose intention is to highlight its possibilities and contribute to a better understanding of this analytical technique. We will not include a systematic review of the T-system (T-Bursts, T-Markers, T-Predictors, T-Retrodictors, ±T-Associates, T-Packets, and T-Composition), because publications regarding these figures are still scarce and so it will be a future aim, although a systematic review of some of these figures has already been carried out (Sáiz-Manzanares et al., 2022).

Having demonstrated the interest contained in this study, the aim is to carry out a systematic review of T-Pattern detection, focused particularly from a methodological perspective.

## 2. Materials and methods

The bibliographical search was carried out in the following databases: SCOPUS, PsycINFO of the American Psychological Association, and Web of Science of Clarivate Analytics (WOS), in line with PRISMA guidelines (Preferred Reporting Items for Systematic Reviews and Meta-Analyses) (Liberati et al., 2009; Moher et al., 2009; Siddaway et al., 2019; Page et al., 2021). The search was performed in title, keywords and abstract; and the general search syntax was: “THEME” AND (“T-Patterns” OR “T Patterns”).

The following inclusion criteria were used, which enabled the application of the corresponding filters: (a) A period from 2000 to 2022; (b) articles published in scientific journals; (c) empirical studies; (d) the thematic areas of Psychology, Behavioral Sciences, and Sport Sciences; (e) English or Spanish languages; (f) access to the whole text (open access, access through the institutions of authors, or purchase).

The following exclusion criteria were taken into account: (a) Documents whose content did not conform to either THEME or T-patterns (these terms were used in a different sense to that defined in the previous sections); (b) document published with a double work codification: as if it were articles in the journal *Neuromethods* and book chapter, but are in fact chapters of the work of Magnusson et al. (2016); and (c) articles that focus on T-patterns and THEME, but are conceptual-methodological in nature and not empirical studies nor systematic reviews.

In addition, the references of the first sample of papers were reviewed in order to request new articles that could meet the criteria indicated.

The included works were reviewed in order to codify: (1) general extrinsic characteristics; (2) bibliometric aspects related to recognition within the scientific community; (3) methodological characteristics considering three levels of codification. Those levels of codification were as follows: (3.1) identify the method explicitly declared by the authors in order to identify the studies based on observational methodology; (3.2) when the paper used observational methodology, the main aspect link to the T-pattern analysis is codified based on Guidelines Reporting Evaluations based on Observational Methodology GREOM (Portell et al., 2015); (3.3) in all the papers that characterize the THEME parameters used to detect T-patterns.

The review of each article was carried out independently by two researchers. The degree of initial agreement was calculated with the Cohen’s kappa coefficient (κ = 0.96).

## 3. Results

### 3.1. Study selection

**FIGURE 2:**
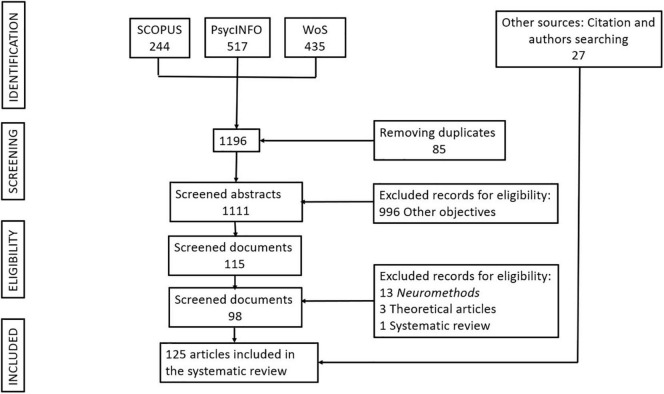
PRISMA diagram.

Figure 2 presents the PRISMA diagram (Page et al., 2021) that shows the selection process of the 125 primary documents that make up this systematic review (see Supplementary Table 1).

### 3.2. Primary document profile

The selected primary documents are of diverse descriptive criteria which we will address here with a view to better clarifying their characteristics.

#### 3.2.1. Extrinsic characteristics of the primary documents

Supplementary Table 2 shows the extrinsic characteristics of the primary documents, and includes information corresponding to: code, authors, number of authors, country of origin, year, research field, and sub-field. It provides a broader view of this scientific production along with highlighting some aspects of it.

**FIGURE 3:**
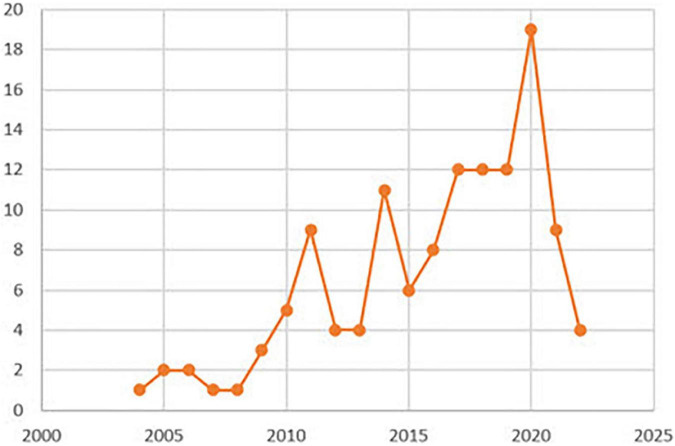
Distribution of primary documents by years.

Publication date (Figure 3) illustrates an increase from 2010, after some anecdotal years, showing a succession of peaks and troughs since then, which, in any case, justifies a consolidation in the use of T-pattern detection analysis.

**FIGURE 4:**
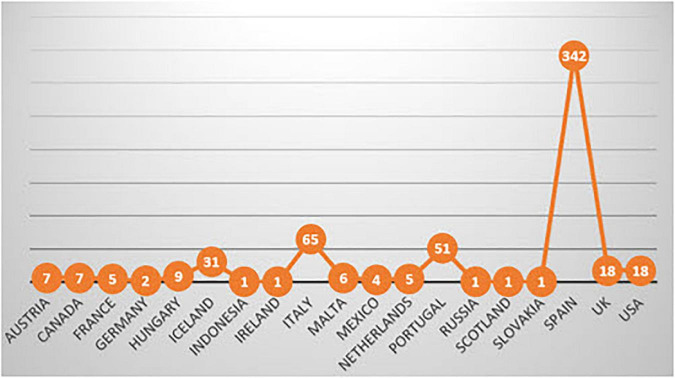
Authors’ countries.

We quantified the number of authors from each publication, and Figure 4 shows the authors’ provenance. Most notably, Spain stands out, with three hundred and forty-two primary documents, well ahead of Italy (65), Portugal (51), and Iceland (31).

**FIGURE 5:**
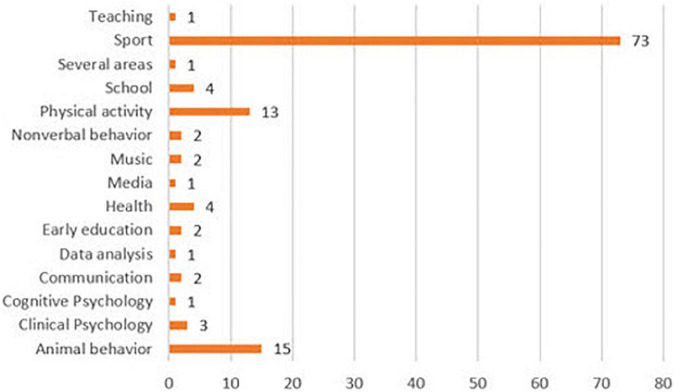
Field.

In terms of the substantive scope (Figure 5), sport is significantly striking, and it has been applied successfully to different sports modalities. Other less prominent fields of study were animal behavior, physical activity, school, and health.

#### 3.2.2. Bibliometric characteristics of the primary documents

Supplementary Table 3 presents the bibliometric characteristics of the primary documents, with the following information: code, authors, database, journal, impact factor, quartile (in accordance with the Web of Science), and quotations.

**FIGURE 6:**
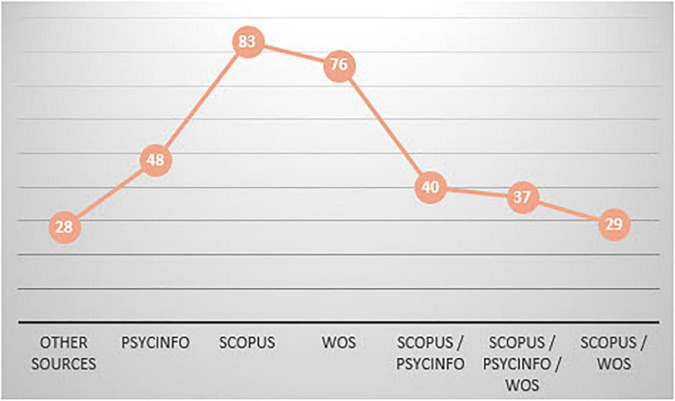
Database.

As previously indicated in the Section “2. Materials and methods,” the primary documents were taken from the SCOPUS, PsycINFO, and WOS databases, in addition to other sources (28 documents, therefore 22.4%) which were accessed from references. We believe it interesting that thirty-seven primary documents (29.6%) were found in the three databases simultaneously (Figure 6).

**FIGURE 7:**
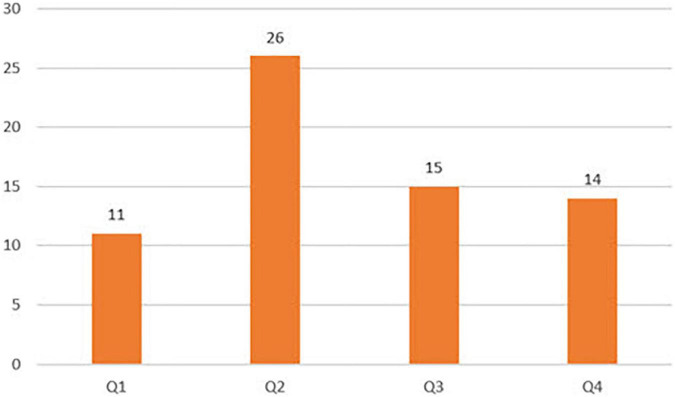
Quartils (from 66 articles).

Given our interest in the scientific quality of the primary documents, we considered it relevant to know whether or not the respective journals —in the years that the documents were published— were included in *Web of Science*. A total of sixty-six articles (52.8%) has an impact factor, with a clear majority of the primary documents (26) being found in quartile 2 (Figure 7).

#### 3.2.3. Methodological characteristics of the primary documents [I]: Data collection, management, data quality control, computer programs, and data analysis

Supplementary Table 4 shows part of the methodological characteristics of the primary documents, providing information about: codes, authors, methodology, design, participants, ethical standards, instrument for collecting data, and number dimensions/categories.

**FIGURE 8:**
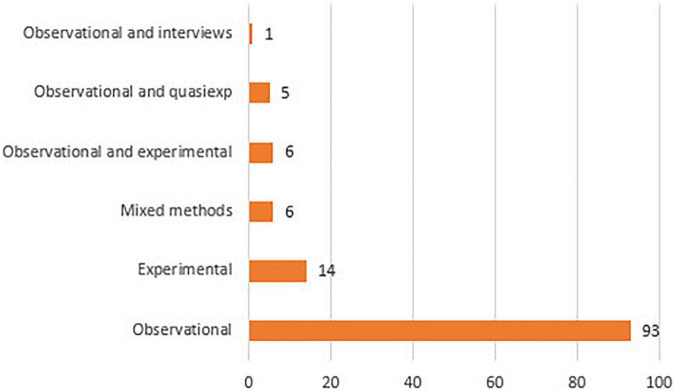
Methodology.

It seems evident that the most repeatedly applied methodology is observational, whether alone (93), or in multi-method studies, in which it is complemented with experimental (6), or with quasi-experimental (5), or with interview (1) (see Figure 8). It is curious that in 6 primary documents the mixed method is explicitly named as the methodology to be applied. Based on previous developments (Anguera and Hernández-Mendo, 2016; Anguera et al., 2017a), we consider that the application of observational methodology implies regarding it as mixed-method in itself. Similarly, we have witnessed the same scenario in indirect observation studies (Anguera et al., 2018).

In Supplementary Table 4 we have included information corresponding to the design, when indicated, which was in 68% of the primary documents. Furthermore, 95.2% of the primary documents specified participant characteristics. The percentage of primary documents that mention ethical standards is lower, at 52%.

In terms of data collection, it is explicitly mentioned in 88% of the primary documents. Due to observational methodology being the most widely applied, there is logically an abundance—84.8%— of made-to-measure (*ad hoc*) instruments. Many of them have been given proper names (SOBL-2, SOCIN, SOPROX, SCOT, SOF5, SOBJUDO-KSGA, OSMOS, SOFEO, SOFCO, ADDEF, OI-INJURIES-FOOTBALL, OTSJUDO, IOUPPERLIMB_FLEX_EXT, OBKA, SINCROBS, ESGRIMOBS, SORPS) or have used an existing proper name (SOF, SOBL, SOFBAS, SOCTM, SsObserWork). The number of dimensions/categories is very heterogeneous.

The methodological characteristics of the primary documents are completed in Supplementary Table 5, including information about computer recording programs, data quality control and the computer programs used, computer programs for data analysis, and data analysis.

In terms of recording programs, whilst being secondary to the aims in this systematic review, out of the a hundred-three studies that specified it, what stands out is the use of LINCE/LINCE PLUS, in 41.6% of the primary documents (records can be directly exported to THEME), whilst the percentage for MATCH VISION STUDIO was 15.2%.

Seventy-two primary documents included data quality control programs, with the majority using GSEQ (48.6%) and LINCE/LINCE PLUS (33.3%).

The use of THEME is inevitable for T-Pattern detection, since it is the only program that allows it. Given that THEME was part of the search syntax, it was obviously used in all the primary studies; however not all the primary documents specified which of the different versions of THEME, available since the year 2000, were used. Among the 31.2% of primary documents who did mention it, the versions used were: THEME 5.0, THEME 6.0, and THEME Edu.

**FIGURE 9:**
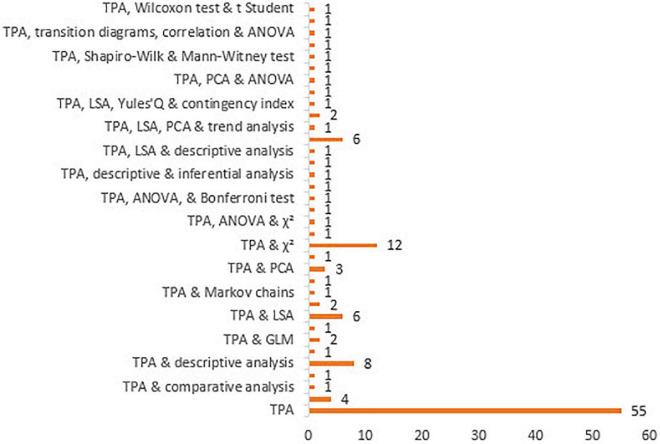
Data analysis.

It is clear that T-pattern detection can be complemented with other analysis techniques, as is shown in Supplementary Table 5 and Figure 9; this being the chosen option for seventy primary documents, notably with the following: χ^2^(11.2%), lag sequential analysis (11.2%), and descriptive analysis (7.2%).

#### 3.2.4. Methodological characteristics of the primary documents (II): T-pattern detection

This forms the study core of this systematic review, which focuses precisely on T-pattern detection (Supplementary Table 6).

We are especially interested in knowing how the primary document search parameters were set. In accordance with the *Reference Manual Pattern Vision Ltd* (2018), decisions are required about: Critical Interval Type, Baseline Probability Type, Minimum Occurrences, Burst Detection, Significance Level, Max Search Levels, Lumping Factor, Exclude Frequent Event Types (Events), Minimum Samples. However, there are no published studies that include information about all of them.

**FIGURE 10:**
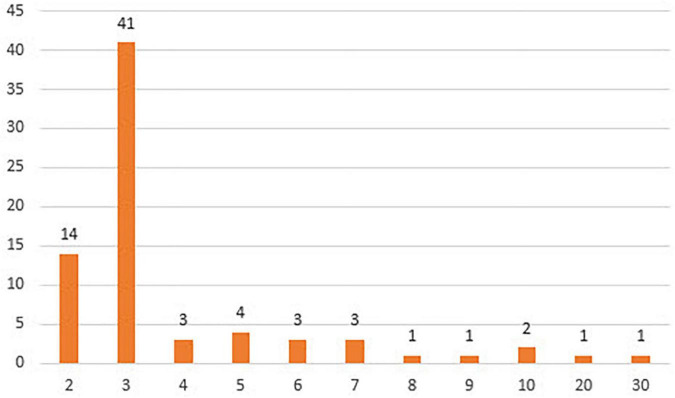
Minimum occurrences.

Firstly, information was collected regarding the *Minimum Occurrences* (with a minimum value of 2) (see Figure 10), with 59.2% of the primary documents containing this information.

Given that some primary documents (22.4%) take into account redundancy reduction (*FARR*) (the recommended value is 90%), it is included in Supplementary Table 6. Furthermore, *randomization* is recommended in order to know whether the detected T-patterns deviate significantly from *random expectation.* The types of *randomization* offered by THEME that some primary documents do mention (28%) are: *shuffling*, *rotation*, and *shuffling and rotation*. We did not include the following parameters in the table: *Minimum samples*, or *FARR*, which usually adopts a standard value of 99 but in some primary documents is different (Wedl et al., 2011 -primary document 123- is 90); *levels of hierarchy*, typical of each database (Wedl et al., 2011 -primary document 123- is 5); *selection of free heuristic critical interval setting* (Pic et al., 2021 -primary document 89-); or “minimum sample,” which can vary greatly depending on the study [is 51 in Casarrubea et al. (2011) -primary document 25-; 100 in Wedl et al. (2011) -primary document 123-].

The number of selected T-Patterns is highly heterogeneous in the primary documents.

In terms of T-pattern selection (15.2% of the studies), the existing options are quantitative, qualitative and structural; moreover, in one of the primary documents (Amatria et al., 2017 -primary document 6-) there is a proposal for qualitative and quantitative filters that was taken into account in later works.

A massive 92% of the primary documents present results, and we have included basic information. Among the results, 15 use tables, 29 use figures, 47 use tables and figures, 10 use tables and figures with incorporated photographs, 1 uses figures with diagrams, 1 uses figures with drawings, and 1 uses tables with photographs.

## 4. Discussion and conclusion

The discovery of hidden patterns in behavior is a task frequently faced by numerous researchers across many investigation areas, e.g., biology, psychology, psychiatry, sport science, robotics, finances, etc. But discovering such patterns has proven to be a challenging task due to a lack of three key matters: first of all, adequate formalized models of the kinds of patterns to look for; secondly, corresponding detection algorithms and, last but not least, their implementation in available software. Over the last decades, these obstacles have been progressively overcome as a result of the introduction of the mathematical T-pattern model and the continued improvement of a technique known as T-pattern detection and analysis (TPA). Several recently published papers have addressed the concepts and examples concerning the applications of TPA in the study of behavior both in human and non-human subjects (Casarrubea et al., 2015, 2018, 2022), that could, together with this systematic review, assist beginners in TPA methodology striving to gain an overview of TPA research.

We highlight the relevance of T-pattern detection in the broad spectrum of fields and sub-fields covered in this systematic review. The detection of structures in behavioral records forms the common nexus between studies carried out in very diverse variants and fields; whether it be from human participants (with highly diversified characteristics, in very different contexts, and analyzing the relationship with behavior, hormone levels, personality, culture, etc.); or animals (dogs, cats, rats, starlings, chickens…); or in studies about the interaction between hormones and behavior; or from movements involved in an individual’s facial expressions, to extensive migratory movements in the marine environment.

A necessary demarcation, as we have justified since our seminal work on observational methodology, is the difference between the use of observation as a method or as a technique (Anguera, 1979, 2003). This difference is well-illustrated in the papers reviewed. They are mainly works that use observation as a method, although T-pattern detection is also used in experimental studies carried out in laboratories, in which observation plays a merely technical role. One cornerstone element is the observation of visually —or even acoustically— perceptible events or behaviors, that are nearly always organized in clusters, and which on many occasions correspond to interactive situations.

Regarding the type of observational design used by studies that apply THEME (I/P/U, I/P/M, I/F/U, I/F/M, N/P/U, N/F/M) —these initials correspond, respectively, to the observational designs Idiographic/Punctual/Unidimensional, Idiographic/Punctual/Multidimensional, Idiographic/Follow-up/Unidimensional/, Idiographic/Follow-up/Multidimensional, Nomothetic/Punctual/Unidimensional, and Nomothetic/Follow-up/Multidimensional- (Anguera et al., 2001; Sánchez-Algarra and Anguera, 2013), it is interesting to highlight that the design is multidimensional in every case (although in two of them the authors define it as one-dimensional). This is consistent with the interest in the use of THEME for the analysis of concurrences, not only between behaviors but also between behaviors and other elements within the context. There is more variability in the other two characteristics of the observational design—idiographic/nomothetic and punctual/follow-up. One element that should be taken into account is that in cases where the design includes just one session, it is intra-sessional monitoring that is analyzed with THEME.

Due to our interest in the methodological aspects that we feel enrich a systematic review, we are aware that in this particular review there are primary documents of varying quality, as can be seen in Supplementary Tables 3–6. For this reason we decided to find out the impact indexes of those which have it (Supplementary Table 3), with a view to identifying those primary documents which are formally considered of better quality.

Whilst THEME appears to be sensitive to low frequency T-patterns, the greatest challenge for the researcher lies in interpreting the results. In most of the studies not all the T-patterns are interpreted, although there are primary studies in which they are interpreted in terms of their growing length (number of successive codes implicated).

While the main contribution of the THEME program is the detection of temporal patterns, it is also possible to detect regular and hidden behavioral patterns depending on the order parameter; and from the assignation of a constant duration to each unit of behavior, which supposes new possibilities for sequential data analysis (Lapresa et al., 2013a,b). There are a number of prominent studies (Alsasua et al., 2018 -primary document 3-; Amatria et al., 2019 -primary document 7-; Alsasua et al., 2021 -primary document 2-) in which T-pattern selection is carried out in accordance with multi-events that show a sequence of consecutive behaviors which make up a specific action. These are identified by the T-patterns themselves (for example, a shot on goal or an attacking tactic in soccer, or a basketball shot), and show efficient-type sequential examples.

Similarly, we would like to highlight that T-pattern detection has been successfully used to differentiate individuals with stereotypes or atypical behavior (Brilot et al., 2009 -primary document 14-), as well as certain profiles of psychiatric patients.

Some primary documents (Burgoon et al., 2014 -primary document 15-) do not only emphasize that T-pattern detection confirms the regularities that show up in behavioral sequences, but they also highlight the role of THEME in discovering patterns that remain “hidden.” Ultimately, the strength of T-pattern detection lies in its ability to localize the connections between temporally related events —although not necessarily contiguous— with the aim of identifying combinations of behaviors that make up a pattern-type structure.

We consider it relevant —due to the possibilities it presents—that in different primary documents (Lapresa et al., 2013b -primary document 69-; Tarragó et al., 2015 -primary document 113-) a constant duration (=1) was conventionally assigned to each event-type, using the THEME program v.6 Edu for the detection of regular structures; bearing in mind that the importance of the analysis does not lie in the duration of each one of the behavior chains, nor in the distance between them, but precisely in their internal sequentiality.

Likewise, we highlight the importance of the qualitative and quantitative filters proposed by Amatria et al. (2017) (-primary document 6-), that were taken into account in some of the primary documents (Amatria et al., 2019 -primary document 7-; Lapresa et al., 2018 -primary document 73-).

In many of the primary documents, the T-pattern detection was complemented by other analysis techniques, and this complementarity is considered recommendable, as indicated in Lapresa et al. (2018) (-primary document 73-), for various reasons. Said reasons are as follows: (a) Lag sequential analysis identifies relationships between individual events that make up a multi-event (Bakeman and Quera, 2011), whilst THEME is able to identify significant relationships between multi-events, or clusters (Tarragó et al., 2017 -primary document 114-); (b) Although THEME (v.6 Edu) detects a negative gravity or repulsion zone in the calculations, it can only generate an inhibiting T-taboo structure (Magnusson, 2000, 2005) when the taboo behavior does not occur. We have not found any studies in which T-taboos have been investigated in THEME (v.6 Edu), although they are relatively common in lag sequential analysis studies based on inhibiting relationships (Tarragó et al., 2017 -primary document 114-).

We would also like to underline that in the primary documents containing other T-pattern detection techniques, there is agreement that THEME detected more T-patterns than the regularities detected by other analyses (Alonso-Vega et al., 2022-primarydocument 1-), and, at least, was maintained in one significant correlation (Brilot et al., 2009 -primary document 14-).

Some primary documents (Brilot et al., 2009 -primary document 14-) consider it difficult to validate T-pattern detection when there is a large quantity of data, and their recommendation in these cases is a statistical comparison with random data, with the aim of achieving an objective confirmation of T-pattern significance.

There five primary documents that were considered atypical (Asher et al., 2009 -primary document 12-; Jonsson et al., 2010 -primary document 63-; Casarrubea et al., 2018 -primary document 24-; Hunyadi, 2019 -primary document 59-; Szekrényes, 2019 -primary document 112-), due to them consisting of brief references to different studies.

There are three main conclusions that can be drawn from this systematic review:

Firstly, there is the extraordinary strength and applicability of T-pattern detection. This enables the researcher to go deeper into a robust analysis, which satisfies the integration of the qualitative and quantitative elements that make up the mixed methods *leitmotif*; thus enabling the discovery of the deep, hidden structure that lies beneath the respective databases, regardless of the methodology used in the study they come from. The diverse possibilities that exist in parameter assignation notably increase the options for obtaining results and for their interpretation.

Secondly, there is the greater presence of T-pattern analysis (TPA) in studies using observational methodology, relative to the use of this technique when other research methods are used.

Thirdly, as systematic reviews can create a framework for analyzing primary data, we musts commit to consolidating the methodological analysis of selected works as well, as taking individual and collective responsibility for improving methodological quality of TPA studies, taking advantage of the resources provided by the THEME program. At the heart of TPA is a pattern detection algorithm that has been in use in number of different scientific fields for over 30 years, were future improvements will deliver more advanced display of results, data import/export, parallel processing, and faster pattern detection.

## Data availability statement

The original contributions presented in this study are included in this article/Supplementary material, further inquiries can be directed to the corresponding author.

## Author contributions

All authors listed have made a substantial, direct, and intellectual contribution to the work, and approved it for publication.
