# Supplementary material for: T-pattern detection in the scientific literature of this century: A systematic review
Source: Front Psychol. 2023 Mar 1;14:1085980. doi: 10.3389/fpsyg.2023.1085980 (PMC10015708; doi:10.3389/fpsyg.2023.1085980)
Supplement: Supplementary file 1 [file Table_1.pdf]

Table 1. Primary documents

1	Alonso-Vega, J., Andrés-López, N., & Froxán-Parga, M.X. (2022). Verbal interaction pattern analysis in clinical psychology. *Frontiers in Psychology,*13:949733. doi: 10.3389/fpsyg.2022.949733
2	Alsasua, R., Arroyo, R., Arana, J., Lapresa, D., & Anguera, M.T. (2021). Influence of the functional class of the players in wheelchair basketball: a comparative match analysis. *Journal of Physical Education and Sport, 21*(6), 3483-3495
3	Alsasua, R., Lapresa, D., Arana, J., Anguera, M.T. y Garzón, B. (2018). Successful and unsuccessful offensive sequences ending in a shot in professional and elite under-16 basketball. *Journal of Human Kinetics, 64*, 147-159. doi: 10.1515/hukin-2017-0191
4	Alves, S.M., Castañer, M., Camerino, O., Franco, S., & Fernandes Rodrigues, J.J. (2016). Comunicação Cinésica e Proxémica de Instrutores de Localizada Experientes e Estagiários: Deteção de T-patterns. *Motricidade, 11*(4), SCOPUS47-57. doi: 10.6063/motricidade.3817
5	Alves, S., Franco, S., Castañer, M., Camerino, O., Rodrigues, J., & Hileno, R. (2015).El análisis de la comunicación paraverbal cinésica y proxémica de los instructores de fitness mediante patrones temporales (T-patterns). *Cuadernos de Psicología del Deporte,15*(1), 111-122
6	Amatria, M., Lapresa, D., Arana, J., Anguera, M.T., & Jonsson, G.K. (2017). Detection and selection of behavioral patterns using Theme: a concrete example in grassroots soccer. *Sports, 5*, 20; doi: 10.3390/sports5010020.
7	Amatria, M., Maneiro, R., & Anguera, M.T. (2019). Analysis of successful offensive play patterns by the Spanish soccer team. *Journal of Human Kinetics, 69*, 191-200. doi: 10.2478/hukin-2019-0011
8	Aragón, S., Lapresa, D., Arana, J., Anguera, M.T. & Garzón, B. (2016). Tactical behaviour of winning athletes in major championship 1500-m and 5000-m track finals. *European Journal of Sport Science, 16*(3), 279-286. doi: 10.1080/17461391.2015.1009494
9	Arbulu, A., Lapresa, D., Usabiaga, O., & Castellano, J. (2016). Detección y aplicación de T-Patterns en la escalada de élite/Detection and application of T-Patterns in elite climbing/Detecção e aplicação de T-Patterns em escalada elite. *Cuadernos de Psicología del Deporte, 16*(1), 95-102.
10	Arias-Pujol, E. & Anguera, M.T. (2020). A Mixed Methods Framework for Psychoanalytic Group Therapy: From Qualitative Records to a Quantitative Approach Using T-Pattern, Lag Sequential and Polar Coordinate Analyses. *Frontiers in Psychology, 11*:1922. doi: 10.3389/fpsyg.2020.01922
11	Argibay-González, J.C., Vázquez-Estévez, C., Gutiérrez-Santiago, A., Paramés-González, A., Reguera-López-de-la-Osa, X., Prieto-Lage, I. (2022). Analysis of Injury Patterns in Men's Football between the English League and the Spanish League. *International Journal of Environmental Research and Public Health, 9*, 11296. doi: 10.3390/ijerph191811296
12	Asher, L., Collins, L.M., Ortiz-Peláez, A., Drewe, J.A., Nicol, C.J., & Pfeiffer, D.U. (2009). Recent advances in the analysis of behavioural organization and interpretation as indicators of animal welfare. *Interface. Journal of the Royal Society, 6*, 1103-1119. doi: 10.1098/rsif.2009.0221
13	Brill, M.& Schwab, F. (2019). A Mixed-Methods Approach Using Self-Report, Observational Time Series Data, and Content Analysis for Process Analysis of a Media Reception Phenomenon. *Frontiers of Psychology, 10:1666.* doi: *10.3389/fpsyg.2019.01666*

14	Brilot, B.O., Asher, L., Feenders, G., & Bateson, M. (2009). Quantification of abnormal repetitive behavior in captive European starlings (Sturnos vulgaris). *Behavioural Processes, 82*(3), 256-264
15	Burgoon, J. K., Proudfoot, J.G., Schuetzler, R., & Wilson, D (2014). Patterns of Nonverbal Behavior Associated with Truth and Deception: Illustrations from Three Experiments**.** *Journal of Nonverbal Behavior*, *38*(3), 325-354.
16	Camerino, L., Camerino, O., Prat, Q., Jonsson, G.K., & Castañer, M. (2020). Has the use of body image in advertising changed in the first two decades of the new century? *Physiology & Behavior, 220,* 112869
17	Camerino, O., Chaverri, J., Anguera, M.T. & Jonsson, G. (2012). Dynamics of the game in soccer: Detection of t-patterns. *European Journal of Sport Science, 12*(3), 216-224.
18	Camerino, O., Prieto, I., Lapresa, D., Gutiérrez-Santiago, A., Hileno, R. (2014). T-pattern detection in combat sports. *Revista de Psicologia del Deporte, 23*(1), 147-155.
19	Camerino, O., Valero-Valenzuela, A., Prat, Q., Manzano Sánchez, D. & Castañer, M. (2019). Optimizing Education: A Mixed Methods Approach Oriented to Teaching Personal and Social Responsibility (TPSR). *Frontiers of Psychology, 10*:1439. doi: 10.3389/fpsyg.2019.01439
20	Casarrubea, M., Aiello, S., Sanangelo, A., Di Giovanni, G., & Crescimanno, G. (2019). Different representation procedures originated from multivariate temporal pattern analysis on the behavioral response to pain in Wistar rats tested in a hot-plate under morphine. *Brain Sciences, 9*, 233.
21	Casarrubea, M, Davies, C., Pierucci, M., Colangeli, R., Deidda, G., Santangfelo, A., Aiello, S., Crescimanno, G., & Di Giovanni, G. (2021). The impact of chronic daily nicotine exposure and its overnight withdrawal on the structure of anxiety-related behaviors in rats: Role of the lateral habenula. *Progress in Neuropsychopharmacology & Biological Psychiatry, 105*, 110131.
22	Casarrubea, M., Di Giovanni, G., & Crescimanno, G. (2021). Effects of different anxiety levels on the behavioral patternings investigated through T-pattern analysis in Wistar rats tested in the hole-board apparatus. *Brain Sciences, 11*, 714. doi: 10.3390/brainsci11060714
23	Casarrubea, M., Faulisi, F., Cudia, A., Cancemi, D., Cardaci, M., Magnusson, M.S., & Crescimanno, G. (2017). Discovery of recurring behavioral sequences in Wistar rat social activity: Possible support to studies on Autism Spectrum Disorders. *Neuroscience Letters, 653*, 58-63.
24	Casarrubea, M., Magnusson, M.S., Anguera, M.T., Jonsson, G.K., Castañer, M., Santangelo, A., Palacino, M., Faulisi, F., Raso, G., Puigarnau, S., Camerino, O., Di Giovanni, G., & Crescimanno, G. (2018). T-pattern detection and analysis for the discovery of hidden features of behaviour. *Journal of Neuroscience Methods, 310*, 24-32. doi: 10.1016/j.jneumeth.2018.06.013
25	Casarrubea, M., Sorbera, F., Magnusson, M.S, & Crescimanno, G. (2011). T-pattern analysis of diazepam-induced modifications on the temporal organization of rat behavioral response to anxiety in hole board. *Psychopharmacology, 215* (1), 177-189
26	Castañer, M., Aiello, S., Prat, Q., Andueza, J., Crescimanno, G., & Camerino, O. (2020). Impulsivity and physical activity: A T-Pattern detection of motor behavior profiles. *Physiology & Behavior, 219*, 112849. doi: 10.1016/j.physbeh.2020.112849
27	Castañer, M., Barreira, D., Camerino, O., Anguera, M.T., Fernandes, T., & Hileno, R. (2017). Mastery in goal scoring, T-pattern detection and polar coordinate analysis of motor skills used by Lionel Messi and Cristiano Ronaldo. *Frontiers in Psychology, 8*:741. doi: 10.3389/fpsyg.2017.00741.

28	Castañer, M., Camerino, O., Anguera, M.T. & Jonsson, G.K. (2010). Observing the paraverbal communicative style of expert and novice PE teachers by means of SOCOP: a sequential analysis. *Procedia - Social and Behavioral Sciences. Innovation and reactivity in Education, 2*(2), 5162–5167.
29	Castañer, M., Camerino, O., Anguera, M.T & Jonsson, G.K. (2013) Kinesics and proxemics communication of expert and novice PE teachers. *Quality & Quantity, 47*(4), 1813-1829.
30	Castañer, M., Saüch, G., Prat, Q., Camerino, O., y Anguera, M.T. (2016). La percepción de beneficios y de la mejora del equilibrio motriz en la actividad física en la tercera edad. *Cuadernos de Psicología del Deporte, 16*(1), 77-84.
31	Cavalera, C., Diana, B., Elia, M., Jonsson, G.K., Zurloni, V. & Anguera, M.T. (2015). T-patterns analysis in soccer games: Relationship between time and attack actions. *Cuadernos de Psicología del Deporte, 15*(1), 41-50.
32	Cenni, C., Casarrubea, M., Gunst, N., Vasey, P.L., Pellis, S.M., Wandia, N., & Leca, J.-B. (2020). Inferring functional patterns of tool use behavior from the temporal structure of object play sequences in a non-human primate species. *Physiology & Behavior, 222,* 112938. doi: 10.1016/j.physbeh.2020.112938.
33	Chaverri, J., Camerino, O., Anguera, M.T., Blanco-Villaseñor, Á. & Losada, J.L. (2010). Interaction contexts in soccer: Detection of T-Patterns. *Gymnasium. Revista de Educação Física, Desporto e Saúde, 2*(1), 69-92.
34	Conceição, A., Silva, A.J., Barbosa, T., Campaniço, J., Costa, A., & Louro, H. (2019). Neuromuscular and motor patterns in breaststroke technique [Padrões motores e musculares na técnica de bruços]. *Revista Brasileira de Cineantropometria e Desempenho Humano, 21*, e56408
35	De Haas, R., Nijdam, A., Westra, T.A., Kas, M.J.H., & Westenberg, H.G.M. (2011). Behavioral pattern analysis and dopamine release in quinpirole-induced repetitive behavior in rats. *Journal of Psychopharmacology, 25*(12), 1712-1719.
36	Diana, B., Zurloni, V., Elia, M., Cavalera, C.M., Jonsson, G.K., & Anguera, M.T. (2017). How game location affects soccer performance: T-pattern analysis of attack actions in home and away matches. *Frontiers in Psychology, 8*:1415. doi: 10.3389/fpsyg.2017.01415
37	Diana, B., Zurloni, V., Elia, M., Cavalera, C., Realdon, O., Jonsson, G.K., & Anguera, M.T. (2018). T-pattern analysis and cognitive load manipulation to detect low-stake lies: an exploratory study. *Frontiers in Psychology, 9*:257, doi: 10.3389/fpsyg.2018.00257.
38	Díaz-Aroca, A. & Arias-Estero, J.L. (2020). Analysis of the free throw technique in formative basketball. *Cuadernos de Psicología del Deporte, 20*(3), 95-108.
39	Escolano-Pérez, E. (2020). Intra-and inter-group differences in the cognitive skills of toddler twins with birth weight discordance: the need to enhance their future from early education. *Sustainability, 12*(24), 1-29. doi: 10.3390/su122410529
40	Escolano-Pérez, E., Herrero-Nivela, M.L., & Anguera, M.T. (2019). Preschool metacognitive skill assessment in order to promote educational sensitive response from mixed-methods approach: complementarity of data analysis. *Frontiers in Psychology, 10*:1298. doi: 10.3389/fpsyg.2019.01298
41	Fernández-Hermógenes, D., Camerino, O., García De Alcaraz, A. (2017). Set-piece offensive plays in soccer [Acciones ofensivas a balón parado en el fútbol]. *Apunts. Educacion Fisica y Deportes, 129*, 78-94.
42	Fernández-Hermógenes, D., Camerino, O. & Hileno, R. (2021). Indicators of Corner Kick Performance in Elite Soccer. *Apunts. Educación Física y Deportes, 144*, 52-64. doi: 10.5672/apunts.2014-0983.es.(2021/2).144.07
43	García-Fariña, A., Jiménez Jiménez, F. y Anguera, M.T. (2016). Análisis observacional del discurso docente del profesorado de educación física a

	través de patrones comunicativos. *Cuadernos de Psicología de Deporte, 16*(1), 171-182.
44	Garzón, B., Lapresa, D., Anguera, M.T. y Arana, J. (2011). Análisis observacional del lanzamiento de tiro libre en jugadores de baloncesto base. *Psicothema, 23*(4), 851-857.
45	Garzón, B., Lapresa, D., Anguera, M.T. y Arana, J. (2014a). Del minibasket al baloncesto: Efectos de la actual configuración reglamentaria en el patrón técnico de lanzamiento de tiro libre. *Revista de Psicología del Deporte, 23*(1), 77-85.
46	Garzón, B., Lapresa, D., Anguera, M.T. y Arana, J. (2014b). Estudio de propuestas intermedias de baloncesto en categoría infantil. *Revista Iberoamericana de Ciencias de la Actividad Física y el Deporte, 1*(1), 10-21.
47	Gunst, N., Casarrubea, M., Vasey, P.L., & Leca, J.-B. (2020). Is female-male mounting functional? An analysis of the temporal patterns of sexual behaviors in Japanese macaques. *Physiology & Behavior, 223*, 112983. doi: 10.1016/j.physbeh.2020.112983
48	Gutiérrez-Santiago, A., Cancela, J.M., Zubiaur, M., & Ayán, C. (2012). Are male judokas with visual impairments training properly? Findings from an observational study. *Journal of Visual Impairment & Blindness, 106*(4), 224-23.
49	Gutiérrez-Santiago, A., Paramés-González, A., & Prieto-Lage, I. (2022). Effect of teaching method on exercise execution in adolescents' use of outdoor fitness equipment. *Perceptual and Motor Skills,129*(4), 1302-1320.
50	Gutierrez-Santiago, A., Gentico-Merino, L.A., & Prieto-Lage, I. (2019). Detection of the technical-tactical pattern of thescoring actions in judo in the men's category of -73 kg. *International Journal of Performance Analysis in Sport, 19*(5), 778-793. doi: 10.1080/24748668.2019.1655934
51	Gutiérrez-Santiago, A., Pereira-Rodríguez, R., & Prieto-Lage, I. (2020). Detection of the technical and tactical motion of the scorable movements in taekwondo. *Physiology & Behavior, 217*,112813. doi: 10.1016/j.physbeh.2020.112813
52	Gutiérrez-Santiago, A., Prieto, I., Ayán, C., & Cancela, J.M. (2013). T-Pattern Detection in Judo Combat: An Approach to Training Male Judokas with Visual Impairments According to Their Weight Category. *International Journal ofSports Science & Coaching, 8*(2), 385-394.
53	Gutiérrez-Santiago, A., Prieto, I., Camerino, O., & Anguera, M.T. (2011a). The temporal structure of judo bouts in visually impaired men and women. *Journal of Sports Sciences, 29*(13), 1443-1451.
54	Gutiérrez-Santiago, A., Prieto, I., Camerino, O. y Anguera, M.T. (2011b). Identificación y análisis del aprendizaje del judo mediante la metodología observacional. *Apunts. Educación Física y Deportes, 104*(2), 46-55.
55	Gutiérrez-Santiago, A., Prieto, I., Camerino, O., & Anguera, M.T. (2012). Sequences of errors in the Judo throw Morote Seoi Nage and their relationship to the learning process. *Proceedings of the Institution of Mechanical Engineers, Part P: Journal of Sports Engineering and Technology, 227*(1), pp. 57-63.
56	Gutiérrez-Santiago, A., Prieto, I., & Cancela, J.M. (2009). Most frequent errors in judo Uki Goshi Technique and the existing relations amont them analysed through T-Patterns. *Journal of Sports Science & Medicine*, 1(8), 36-46.
57	Gutiérrez-Santiago, A., Prieto, I., Cancela, J., & Ayán, C. (2014). Análisis del error en la técnica de judo koshi guruma mediante t-patterns. *Revista Internacional de Medicina y Ciencias de la Actividad Física y del Deporte, 14*(55), 393-407.
58	Hocking, P.M., Rutherford, K.M.D., & Picard, M. (2007). Comparison of time-based frequencies, fractal análisis and T-patterns for assessing behavioural

	changes in broiler breeders fedon two diets at two levels of feed restriction: A case study. *Applied Animal Behaviour Science, 104*(1-2), 37-48.
59	Hunyadi, L. (2019). Agreeing/disagreeing in a dialogue: Multimodal patterns of its expression. *Frontiers in Psychology, 10*:1373-1373.
60	Ibáñez, R., Lapresa, D., Arana, J., Camerino, O., & Anguera, M.T. (2018). Observational Analysis of the Technical-Tactical Performance of Elite Karate Contestants. *Ciencia, Cultura y Deporte, 13*, 61-70.
61	Iglesias, X., Rodríguez-Zamora, L., Chaverri, D., Clapés, P., Rodríguez, F.A. y Anguera, M.T. (2015). Diversificación de patrones en rutinas de solo en natación sincronizada de alto nivel. *Cuadernos de Psicología del Deporte, 15*(1), 89-98.
62	Jonsson, G.K., Anguera, M.T., Blanco-Villaseñor, A., Losada, J.L., Hernández-Mendo, A., Ardá, T., Camerino, O. & Castellano, J. (2006). Hidden patterns of play interaction in soccer using SOF-CODER. *Behavior Research Methods, 38*(3), 372-381. doi: 10.3758/BF03192790.
63	Jonsson, G.K., Anguera, M.T., Sánchez-Algarra, P., Oliveira, C., Campaniço, J., Castañer, M., Torrents, C., Dinusová, M.., Chaverri, J.., Camerino, O., & Magnusson, M.S. (2010). Application of T-Pattern Detection and Analysis in Sports Research. *The Open Sports Sciences Journal, 3*, 62-71.
64	Kemp, A.S., Fillmore, P.T., Lenjavi, M.R., (…), Touchette, P.E., & Sandman, C.A. (2008). Temporal patterns of self-injurious behavior correlate with stress hormone levels in the developmentally disabled. *Psychiatry Research, 157*(1-3), 181-189.
65	Kerepesi, A., Jonsson, G.K., Mikloso, Á., Topál, J., Csányi, V., & Magnusson, M.S. (2005). Detection of temporal patterns in dog-human interaction. *Behavioural Processes, 70*, 69-79.
66	Kerepesi, A., Kubinyi, E., Jonsson, G.K., Magnusson, M.S., & Miklósi, A. (2006). Behavioural comparison of human-animal (dog) and human-robot (AIBO) interactions. *Behavioural Processes, 73*(1), 92-99.
67	Lapresa, D., Alsasua, R., Arana, J., Anguera, M.T. y Garzón, B. (2014). Análisis observacional de la construcción de las secuencias ofensivas que acaban en lanzamiento en baloncesto de categoría infantil. *Revista de Psicología del Deporte, 23*(2), 365-376.
68	Lapresa, D., Anguera, M.T., Alsasua, R., Arana, J. & Garzón, B. (2013). Comparative analysis of T-patterns using real time data and simulated data by assignment of conventional durations: the construction of efficacy in children's basketball. *International Journal of Performance Analysis in Sport, 13*, 321-339.
69	Lapresa, D., Arana, J., Anguera, M.T. & Garzón, B. (2013). Comparative analysis of the sequentiality using SDIS-GSEQ and THEME: a concrete example in soccer. *Journal of Sports Sciences, 31*(15), 1687-1695.
70	Lapresa, D., Blanco, F., Amatria, M., Arana, J., & Anguera, M. T. (2020). Observational Analysis of the Execution of the "Control" Core Technical/Tactical Concept by Sergio Busquets. *Apunts. Educación Física y Deportes*, 140, 52-62. doi: 10.5672/apunts.2014-0983.es.(2020/2).140.08
71	Lapresa, D., Camerino, O., Cabedo, J., Anguera, M.T., Jonsson, G.K. y Arana, X. (2015). Degradación de *T-patterns* en estudios observacionales: Un estudio sobre la eficacia en el ataque de fútbol sala. *Cuadernos de Psicología del Deporte, 15*(1), 71-82.
72	Lapresa, D., Chivite, J., Arana, J., Anguera, M.T., y Barbero, J.R. (2018). Análisis de la eficacia del portero de fútbol cadete (14-16 años). *Apunts. Educación Física y Deportes, 131*(1), 60-79. doi: 10.5672/apunts.2014-0983.es.(2018/1).131.05.

73	Lapresa, D., Del Río, A., Arana, J., Amatria, M., & Anguera, M.T. (2018). Use of effective play-space by U12 FC Barcelona players: an observational study combining lag sequential analysis and T-pattern detection. *International Journal of Performance Analysis in Sport, 18*(2), 293-309. doi: 10.1080/24748668.2018.1475195.
74	Lapresa, D., García Pascual, J., Arana, J., & Garzón, B. (2011). Analysis of time patterns in preshooting gestural routines in basketball free-throw shooting by children 11 to 12 years old. *Revista de Psicologia del Deporte, 20*(2), 383-400.
75	Lapresa, D., Ibáñez, R., Arana, J., Garzón, B., Amatria, M. (2011). Spatial and temporal analysis of Karate Kumite moves: Comparative study of the senior and 12-13 year old groups. *International Journal of Performance Analysis in Sport, 11*(1), 57-70.
76	Lapresa, D., Pascual, J., Arana, J., y Anguera, M.T. (2019). Sistema de observación para analizar la interacción en el juego de Boccia por equipos. *Cuadernos de Psicología del Deporte, 20*(1), 37-47.
77	Lapresa, D., Santesteban, G., Arana, J., Anguera, M.T., & Aragón, S. (2019). Observation System for Analyzing Individual Boccia BC3. *Journal of Development and Physical Disabilities, 29*, 721-734. doi: 10.1007/s10882-017-9552-2.
78	Lapresa, D., Solano, R., Arana, J., Anguera, M.T., y Aragón, S. (2018). Estudio observacional de la salida de tacos de atletismo en las fases especificas "a sus puestos" y "listos". *Revista Iberoamericana de Psicología del Ejercicio y el Deporte, 13*(1), 145-153.
79	Lavega-Burgués, P., Luchoro-Parrilla, R.A., Serna, J., Salas-Santandreu, C., Aires-Araujo, P., Rodriguez-Arregi, R., Muñoz-Arroyave, V., Ensenyat, A., Damian-Silva, S., Machado, L., Prat, Q., Sáez de Ocáriz, U., Rillo-Albert, A., Martín-Martínez, D., Pic, M. (2020). Enhancing Multimodal Learning Through Traditional Sporting Games: Marro360°. *Frontiers in Psychology, 11*:1384
80	Louro, H., Silva, A. J., Anguera, M. T., Marinho, D. A., Oliveira, C., Conceição, A. & Campaniço, J. (2010). Stability of patterns of behavior in the butterfly technique of the elite swimmers. *Journal of Sports Science and Medicine, 9*, 36-50.
81	Lyon, M. & Kemp, A.S. (2004). Increased temporal patterns in choice responding and altered cognitive processes in schizophrenia and mania. *Psychopharmacology, 172*(2), 211-219.
82	Merlet, F., Puterflam, J., Faure, J.M., Hocking, P.M., Magnusson, M.S., & Picard, M. (2005). Detection and comparison of time patterns of behaviours of two brooler breeder genotypes fed ad libitum and two levels of fed restriction. *Applied Animal Behaviour Science, 94*(3-4), 255-271.
83	Pic, M. (2017). Different motor patterns in basketball depending on gender?. *Cuadernos de Psicologia del Deporte, 17*(3), 149-156.
84	Pic, M. (2017). The observation of gender differences in handball. *E-Balonmano.Com: Revista de Ciencias del Deporte, 13*(3), 191-198
85	Pic, M. (2018a). Temporal consistencies in two champion teams of European football? *Retos, 34*, 94-99.
86	Pic, M. (2018b). Performance and Home Advantage in Handball. *Journal of Human Kinetics, 63*(1), 61-71.
87	Pic, M. & Jonsson, G.K. (2021). Professional boxing analysis with T-Patterns. *Physiology & Behavior*, *232,*113329
88	Pic, M., Navarro-Adelantado, V., & Jonsson, G. K. (2018). Detection of Ludic Patterns in Two Triadic Motor Games and Differences in Decision Complexity. *Frontiers in Psychology, 8*:2259.

89	Pic, M., Navarro-Adelantado, V., & Jonsson, G.K. (2021). Exploring playful asymmetries for gender-related decision-making through T-pattern analysis. *Physiology & Behavior, 236*, 113421-113421
90	Portell, M., Sene-Mir, A.M., Anguera, M.T., Jonsson, G.K., & Losada, J.L. (2019). Support system for the assessment and intervention during the manual material handling training at the workplace: Contributions from the systematic observation. *Frontiers in Psychology 10*:1247. doi: 10.3389/fpsyg.2019.01247
91	Prat, Q., Andueza, J., Echávarri, Camerino, O., Fernandes, T., & Castañer, M. (2019). A mixed methods design to detect adolescent and young adults' impulsiveness on decision-making and motor performance. *Frontiers in Psychology, 10*:1072. doi: 10.3389/fpsyg.2019.01072
92	Prieto-Lage, I., Artigues-Ribas, L., Gutiérrez-Santiago, A. (2020). Patrones técnico-tácticos del lanzador y el portero en los penales de la liga española de fútbol durante la temporada 2016-17 mediante *T-patterns* y coordenadas polares. *Cuadernos de Psicología del Deporte, 20*(1), 166-180.
93	Prieto-Lage, I., Gutiérrez-Santiago, A., Camerino, O. & Anguera, M.T. (2013). Knowledge of error in relation to the teaching and learning osoto-gari judo throw. *International Journal of Sport Sciences & Coaching, 8*(1), 53-62.
94	Prieto-Lage, I., Gutiérrez-Santiago, A., Curran, P.T., Prieto, M.A. (2016). Injury assessment of common nage-waza judo techniques for amateur judokas. *International Journal of Performance in Sport, 16*(3), 961-982. doi: 10.1080/24748668.2016.11868942
95	Prieto-Lage, I., Gutiérrez-Santiago, A., Lage, M.A.P. (2014). Knowledge of errors in the teaching-learning process of judo-techniques: Osoto-guruma as a case study. *Journal of Human Kinetics, 41*(1), 253-263.
96	Prieto-Lage, I., Louzao-Neira, I., Argibay-González, J. C., & Gutiérrez-Santiago, A. (2020). Injury patterns of profesional footballers in the Spanish first division during the 2017-2018 seasons. *Physiology & Behavior, 224*, 113052.
97	Prieto-Lage, I., Rodríguez-Souto, M., Prieto, M. A., Gutiérrez-Santiago, A. (2020). Technical análisis in Tsuri-goshi through three complementary observational analysis. *Physiology & Behavior, 216*, 112804.
98	Sandman, C.A., Kemp, A.S., Mabini, C., Pincus, D., Magnusson, M. (2012). The role of self-injury in the organisation of behaviour. *Journal of Intellectual Disability Research, 56*(5), 516-526.
99	Santangelo, A., Monteleone, A.M., Casarrubea, M., Cassioli, E., Castellini, G., Crescimanno, G., Aiello, S., Ruzzi, V., Cascino, G., Marciello, F., & Ricca, V. (2020), Recurring sequences of multimodal non-verbal and verbal communication during a human psycho-social stress test: A temporal pattern analysis. *Physiology & Behavior, 221*, 112907.
100	Santos, F., Lourenco, J., Sarmento, H., Mendes, B., Mauricio, N., Furtado, B., Sousa, P., & Pinheiro, V. (2017). Análise complementar do golo no futebol através de análise notacional, análise sequencial e deteçao de T-Patterns. *Revista Brasileira de Futsal e Futebol, 9* (34), 238-249.
101	Santos, F., Sarmento, H., Louro, H., Lopes, H.M., & Rodrigues, J.J. (2014). T-patterns detection in competition football coaches**.** *Motricidade, 10*(4), 64-83.
102	Santos, F., Santos, J., Espada, M., Ferreira, C., Sousa, P., & Pinheiro, V. (2022) T-*pattern* analysis of offensive and defensive actions of youth football goalkeepers. *Frontiers in Psychology,* 13:957858. doi: 10.3389/fpsyg.2022.957858
103	Santos, F.J., Vilarigues, I., Silva, C., Figueiredo, T., & Espada, M. (2021). Analysis of instruction in youth training with handball coaches. *Motricidade, 17*(2), 108-118.

104	Santoyo, C., Jonsson, G.K., Anguera, M.T., & López-López, J.A. (2017). Observational Analysis of the Organization of On-Task Behavior in the Classroom Using Complementary Data Analyses. *Anales de Psicología, 33*(3), 497-514.
105	Santoyo, C., Jonsson, G.K., Anguera, M.T., Portell, M., Allegro, A., Colmenares, L., & Torres, G.Y. (2020). T-Patterns integration strategy in a longitudinal study: A multiple case analysis. *Physiology & Behavior, 222*, 112904. doi: 10.1016/j.physbeh.2020.112904
106	Sastre, V., Lapresa, D., Arana, J., Ibáñez, R. & Anguera, M.T. (2021). Observational analysis of technical-tactical performance in initiation to combat in karate. *International Journal of Performance Analysis in Sport, 21*(1), 126-138. Online first. doi: 10.1080/24748668.2020.1853450
107	Sarmento, H., Bradley, P.S., Anguera, M.T., Polido, T., Resende, R., & Campaniço, J. (2015). Quantifying the offensive sequences that result in goals in elite futsal matches. *Journal of Sports Sciences, 34*(7), 621-629. doi: 10.1080/02640414.2015.1066024
108	Saüch, G., Castañer, M. (2014). Observing motor behaviour patterns in exercise programmes for the elderly and an analysis of user views [Observación de patrones motrices generados por los programas de actividad física para la tercera edad y la percepción de sus usuarios]. *Revista de Psicologia del Deporte, 23*(1), 181-190.
109	Sene-Mir, A. M., Portell, M., Anguera, M. T., & Chacón-Moscoso, S. (2020). Manual Material Handling Training: The Effect of Self-Observation, Hetero-Observational and Intrinsic Feedback on Workers' Knowledge and Behaviour. *International Journal of Environmental Research and Public Health, 17*(21), 8095. doi: 10.3390/ijerph17218095
110	Serna-Bardavío, J., Muñoz-Arroyave, V., Hileno, R., Solsona, E., & Sáez de Ocáriz, U. (2017). T-patterns initiated with ball screen or one-on-one in basketball. *Revista de Psicologia del Deporte, 26*, 81-86.
111	Suárez, N., Sánchez-López, C.R., Jiménez, J.E., & Anguera, M.T. (2018). Is reading instruction evidence-based? Analyzing teaching practices using T-Patterns. *Frontiers in Psychology, 9:*7. doi: 10.3389/fpsyg.2018.00007
112	Szekrényes, I. (2019). Post-processing t-patterns using external tools from a mixed method perspective. *Frontiers in Psychology*, 10:1680
113	Tarragó, R., Iglesias, X., Lapresa, D. y Anguera, M.T. (2016). Complementariedad entre las relaciones diacrónicas de los *T-Patterns* y los patrones de conducta en acciones de esgrima de espada masculina de élite. *Cuadernos de Psicología de Deporte, 16*(1), 113-128.
114	Tarragó, R., Iglesias, X., Lapresa, D., Anguera, M.T., Ruiz-Sanchís, L., & Arana, J. (2017). Analysis of diachronic relationships in successful and unsuccessful behaviors by world fencing champions using three complementary techniques. *Anales de Psicología, 33*(3), 471-485.
115	Tarragó, R., Iglesias, X., Michavila, J.J., Chaverri, D., Ruiz-Sanchís, L. y Anguera, M.T. (2015). Análisis de patrones en asaltos de espada de alto nivel. *Cuadernos de Psicología del Deporte, 15*(1), 149-158.
116	Terroba, M., Ribera, J.M., Lapresa, D., & Anguera, M.T. (2021). Education intervention using a ground robot with programmed directional controls: observational analysis of the development of computational thinking in Early Childhood Education. *Revista de Psicodidáctica, 26*(2), 143-151. https://doi.org/10.1016/j.psicod.2021.03.001
117	Torrents, C. Castañer, M. & Anguera, M.T. (2011). Dancing with complexity: Observation of emergent patterns in dance improvisation. *Education, Physical Training, Sport, 80*(1), 76-81.

118	Torrents, C., Castañer, M., Dinušová, M. & Anguera, M.T. (2010). Discovering new ways of moving: Observational analysis of motor creativity while dancing contact improvisation and the influence of the partner. *Journal of Creative Behavior, 44*(1), 53-69.
119	Tripiana, S. (2018). Development of the strategy of fragmented practice or chunking in the piano studio [Implementación de la estrategia de práctica fragmentada o chunking en el aula de piano]. *Estudios Pedagógicos, 44*(3), 193-215.
120	Tripiana, S., & Vela, M. (2020). Análisis musical aplicado a la interpretación como estrategia de práctica instrumental. *Revista Internacional de Educación Musical, 8*, 3-13. doi: 10.1177/2307484120956511
121	Valero-Valenzuela, A., Camerino, O., Manzano-Sánchez, D., Prat, Q., Castañer, M. (2020). Enhancing learner motivation and classroom social climate: A mixed methods approach. *International Journal of Environmental Research and Public Health, 17*, 5272. doi: 10.3390/ijerph17155272
122	Valero-Valenzuela, A., García, D.G., Camerino, O., & Manzano, D. (2020). Hybridisation of the teaching personal and social responsibility model and gamification in physical education. *Apunts. Educacion Fisica y Deportes*, *141*, 63-74.
123	Wedl, M., Bauer, B., Gracey, D., Grabmayer, C.; Spielauer, E., Day, J., & Kotrschal, K. (2011). Factors influencing the temporal patterns of dyadic behaviours and interactions between domestic cats and their owners. *Behavioural Processes, 86*(1), 58-67.
124	Zurloni, V., Cavalera, C., Diana, B., Elia, M., & Jonsson, G.K. (2014). Detección de regularidades en la dinámica del fútbol: Un enfoque desde los T-pattern [Detecting regularities in soccer dynamics: A T-pattern approach]. *Revista de Psicología del Deporte, 23*(1), 157-164.
125	Zurloni, V., Diana, B., Cavalera, C., Argenton, L., Elia, M., & Mantovani, F. (2015). Deceptive behavior in doping related interviews: The case of Lance Armstrong. *Psychology of Sport and Exercise*, *16*(Part 2), 191-200.
