# Supplementary material for: T-pattern detection in the scientific literature of this century: A systematic review
Source: Front Psychol. 2023 Mar 1;14:1085980. doi: 10.3389/fpsyg.2023.1085980 (PMC10015708; doi:10.3389/fpsyg.2023.1085980)
Supplement: Supplementary file 2 [file Table_2.pdf]

Table 2. Extrinsic characteristics of the primary documents

Code	Authors	Number authors	Countries of authors	Year	Research field	Subfield
1	Alonso-Vega et al.	3	Spain (3)	2022	Clinical Psychology	Psychological interventions
2	Alsasua et al.	5	Spain (5)	2021	Sport	Basketball
3	Alsasua et al.	5	Spain (6)	2018	Sport	Basketball
4	Alves et al.	5	Portugal (3) and Spain (2)	2016	Physical activity	Fitness
5	Alves et al.	6	Portugal (3) and Spain (3)	2015	Physical activity	Fitness
6	Amatria et al.	5	Spain (5)	2017	Sport	Soccer
7	Amatria et al.	3	Spain (3)	2019	Sport	Soccer
8	Aragón et al.	5	Spain (5)	2016	Sport	Athletism
9	Arbulu et al.	4	Spain (4)	2016	Sport	Climbing
10	Arias-Pujol et al.	2	Spain (2)	2020	Clinical Psychology	Psychotherapy
11	Argibay et al.	6	Spain (6)	2022	Sport	Soccer
12	Asher et al.	6	UK (6)	2009	Animal behavior	Animal welfare
13	Brill et al.	2	Germany (2)	2019	Media	Media stimulus
14	Brilot et al.	4	UK (4)	2009	Animal behavior	Stereotipies
15	Burgoon et al.	4	USA (4)	2014	Nonverbal behavior	Truth and deception
16	Camerino et al.	5	Spain (4) and Iceland (1)	2020	Nonverbal behavior	Body image
17	Camerino et al.	4	Iceland (1) and Spain (3)	2012	Sport	Soccer
18	Camerino et al.	5	Spain (5)	2014	Sport	Combat
19	Camerino et al.	5	Spain (5)	2019	Physical activity	Teaching
20	Casarrubea et al.	5	Italy (4) and Malta (1)	2019	Animal behavior	Morphine
21	Casarrubea et al.	9	Italy (3), Malta (3), and UK (3)	2021	Animal behavior	Nicotine
22	Casarrubea et al.	3	Italy (2) and Malta (1)	2021	Animal behavior	Anxiety
23	Casarrubea et al.	7	Italy (6) and Iceland (1)	2017	Animal behavior	Autism
24	Casarrubea et al.	13	Italy (7), Iceland (2), Malta (1), and Spain (4)	2018	Several areas	
25	Casarrubea et al.	4	Italy (3) and Iceland (1)	2011	Animal behavior	Diazephan
26	Castañer et al.	6	Spain (4) and Italy (2)	2020	Physical activity	Motor behavior
27	Castañer et al.	6	Portugal (2) and Spain(4)	2017	Sport	Soccer
28	Castañer et al.	4	Iceland (1) and Spain (3)	2010	Physical activity	Teaching
29	Castañer et al.	4	Iceland (1) and Spain (3)	2013	Physical activity	Teaching

30	Castañer et al.	5	Spain (5)	2016	Physical activity	Elderty
31	Cavalera et al.	6	Italy (4), Iceland (1), and Spain (1)	2015	Sport	Soccer
32	Cenni et al.	7	Canada (5), Italy (1), and Indonesia (1)	2020	Anima behavior	Primate species
33	Chaverri et al.	5	Spain (5)	2010	Sport	Soccer
34	Conceição et al.	6	Portugal (6)	2019	Sport	Swimming
35	De Haas et al.	5	Netherlands (5)	2011	Animal behavior	Rats
36	Diana et al.	6	Italy (4), Iceland (1), and Spain (1)	2017	Sport	Soccer
37	Diana et al.	7	Italy (5), Iceland (1), and Spain (1)	2018	Cognitive Psychology	Deception
38	Díaz-Aroca et al.	2	Spain (2)	2020	Sport	Basketball
39	Escolano-Pérez	1	Spain (1)	2020	Early education	Weight discordance
40	Escolano-Pérez et al.	3	Spain (3)	2019	Early education	Educational sensitive response
41	Fernández-Hermógenes et al.	3	Spain (3)	2017	Sport	Soccer
42	Fernández-Hermógenes et al.	3	Spain (3)	2021	Sport	Soccer
43	García-Fariña et al.	3	Spain (3)	2016	Physical activity	Teaching
44	Garzón et al.	4	Spain (4)	2011	Sport	Basketball
45	Garzón et al.	4	Spain (4)	2014a	Sport	Basketball
46	Garzón et al.	4	Spain (4)	2014b	Sport	Basketball
47	Gunst et al.	4	Canada (2) and Italy (2)	2020	Animal behavior	Primate species
48	Gutiérrez-Santiago et al.	4	Spain (4)	2012	Sport	Judo
49	Gutiérrez-Santiago et al.	3	Spain (3)	2022	Sport	Fitness
50	Gutiérrez-Santiago et al.	3	Spain (3)	2019	Sport	Judo
51	Gutiérrez-Santiago et al.	3	Spain (3)	2020	Sport	Taekwondo
52	Gutiérrez-Santiago et al.	4	Spain (4)	2013	Sport	Judo
53	Gutiérrez-Santiago et al.	4	Spain (4)	2011a	Sport	Judo
54	Gutiérrez-Santiago et al.	4	Spain (4)	2011b	Sport	Judo
55	Gutiérrez-Santiago et al.	4	Spain (4)	2012	Sport	Judo
56	Gutiérrez-Santiago et al.	3	Spain (3)	2009	Sport	Judo
57	Gutiérrez-Santiago et al.	4	Spain (3)	2014	Sport	Judo
58	Hocking et al.	3	Scotland (1) and France (1)	2007	Animal behavior	Broiler breeders
59	Hunyadi	1	Hungary (1)	2019	Communication	Multimodality
60	Ibáñez et al.	5	Spain (5)	2018	Sport	Karate
61	Iglesias et al.	6	Spain (6)	2015	Sport	Swimming
62	Jonsson et al.	8	Iceland (1) and Spain (7)	2006	Sport	Soccer
63	Jonsson et al.	11	Iceland (2), Portugal (2) and Spain (7)	2010	Sport	Soccer, boxing, basketball, swimming and motor skill analysis
64	Kemp et al.	7	USA (7)	2008	Health	Self-injurious behavior

65	Kerepesi et al.	6	Hungary (4) and Iceland (2)	2005	Animal behavior	Dog-human interaction
66	Kerepesi et al.	5	Hungary (3) and Iceland (2)	2006	Animal behavior	Behavioural comparison
67	Lapresa et al.	5	Spain (5)	2014	Sport	Basketball
68	Lapresa et al.	5	Spain (5)	2013	Sport	Basketball
69	Lapresa et al.	4	Spain (4)	2013	Sport	Soccer
70	Lapresa et al.	5	Spain (5)	2020	Sport	Soccer
71	Lapresa et al.	6	Iceland (1) and Spain (5)	2015	Sport	Futsal
72	Lapresa et al.	5	Spain (5)	2018	Sport	Soccer
73	Lapresa et al.	5	Spain (5)	2018	Sport	Soccer
74	Lapresa et al.	4	Spain (4)	2011	Sport	Basketball
75	Lapresa et al.	5	Spain (5)	2011	Sport	Karate
76	Lapresa et al.	4	Spain (4)	2019	Sport	Bocchia
77	Lapresa et al.	5	Spain (5)	2019	Sport	Bocchia
78	Lapresa et al.	5	Spain (5)	2018	Sport	Athletism
79	Lavega et al.	15	Russia (1) and Spain (14)	2020	Sport	Games
80	Louro et al.	7	Portugal (6) and Spain (1)	2010	Sport	Swimming
81	Lyon et al.	2	USA (2)	2004	Health	Schizophrenia and mania
82	Merlet et al.	6	France (4), USA (1), and Iceland (1)	2005	Animal behavior	Broiler breeders
83	Pic	1	Spain (1)	2017	Sport	Basketball
84	Pic	1	Spain (1)	2017	Sport	Handball
85	Pic	1	Spain (1)	2018a	Sport	Football
86	Pic	1	Spain (1)	2018b	Sport	Handball
87	Pic et al.	2	Iceland (1) and Spain (1)	2021	Sport	Boxing
88	Pic et al.	3	Iceland (1) and Spain (2)	2018	Sport	Motor games
89	Pic et al.	3	Iceland (1) and Spain (2)	2021	Sport	Motor games
90	Portell et al.	5	Iceland (1) and Spain (4)	2019	Health	Workplace
91	Prat et al.	6	Spain (5) and Portugal (1)	2019	Physical activity	Decision-making and motor performance
92	Prieto-Lage et al.	3	Spain (3)	2020	Sport	Football
93	Prieto-Lage et al.	4	Spain (4)	2013	Sport	Judo
94	Prieto-Lage et al.	4	Spain (3) and Ireland (1)	2016	Sport	Judo
95	Prieto-Lage et al.	3	Spain (3)	2014	Sport	Judo
96	Prieto-Lage et al.	4	Spain (4)	2020	Sport	Football
97	Prieto-Lage et al.	4	Spain (4)	2020	Sport	Judo
98	Sandman et al.	5	USA (4) and Iceland (1)	2012	Clinical Psychology	Organization of behavior
99	Santangelo et al.	11	Italy (11)	2020	Communication	Multiodality

100	Santos et al.	8	Portugal (8)	2017	Sport	Football
101	Santos et al.	5	Portugal (5)	2014	Sport	Football
102	Santos et al.	6	Portugal (6)	2022	Sport	Soccer
103	Santos et al.	5	Portugal (5)	2021	Sport	Handball
104	Santoyo et al.	4	Iceland (1), México (1) and Spain (2)	2017	School	Classroom
105	Santoyo et al.	7	Iceland (1), Italy (1), México (3) and Spain (2)	2020	School	Social interaction
106	Sastre et al.	5	Spain (5)	2021	Sport	Karate
107	Sarmento et al.	6	Portugal (4), UK (1), and Spain (1)	2016	Sport	Futsal
108	Saüch et al.	2	Spain (2)	2014	Psysical activity	Elderty
109	Sene-Mir et al.	4	Spain (4)	2020	Health	Workplace
110	Serna-Bardavío et al.	5	Spain (5)	2017	Sport	Basketball
111	Suárez et al.	4	Spain (4)	2018	School	Primary teachers
112	Szekrényes	1	Hungary (1)	2019	Data analysis	Tool implemented
113	Tarragó et al.	4	Spain (4)	2016	Sport	Fencing
114	Tarragó et al.	6	Spain (6)	2017	Sport	Fencing
115	Tarragó et al.	6	Spain (6)	2015	Sport	Fencing
116	Terroba et al.	4	Spain (4)	2021	School	Early childhood education
117	Torrents et al.	3	Spain (3)	2011	Physical activity	Dance
118	Torrents et al.	4	Spain (3) and Slovenia (1)	2010	Physical activity	Dance
119	Tripiana	1	Spain (1)	2018	Music	Piano
120	Tripiana et al.	2	Spain (2)	2020	Music	16 instrumental specialities
121	Valero et al.	5	Spain (5)	2020	Teaching	Clasroom social climate
122	Valero et al.	4	Spain (4)	2020	Psysical activity	Teaching
123	Wedl et al.	7	Austria (7)	2011	Animal behavior	Domestic cats
124	Zurloni et al.	5	Iceland (1) + Italy (4)	2014	Sport	Soccer
125	Zurloni et al.	6	Italy (6)	2015	Sport	Doping
