# Supplementary material for: T-pattern detection in the scientific literature of this century: A systematic review
Source: Front Psychol. 2023 Mar 1;14:1085980. doi: 10.3389/fpsyg.2023.1085980 (PMC10015708; doi:10.3389/fpsyg.2023.1085980)
Supplement: Supplementary file 3 [file Table_3.pdf]

Table 3. Bibliometric characteristics of the primary documents

Code	Authors	Database	Quotations	Journal	Impact factor	Quartile
1	Alonso-Vega et al.	OTHER SOURCES		Frontiers in Psychology	4,232 (2021)	Q1
2	Alsasua et al.	SCOPUS		Journal of Physical Education and Sport		
3	Alsasua et al.	SCOPUS / WOS	6	Journal of Human Kinetics	1,414	Q3
4	Alves et al.	WOS		Motricidade		
5	Alves et al.	SCOPUS / PSYCINFO / WOS	48	Cuadernos de Psicología del Deporte		
6	Amatria et al.	SCOPUS / WOS	69	Sports	0,23	
7	Amatria et al.	OTHER SOURCES		Journal of Human Kinetics	1,664	Q3
8	Aragón et al.	SCOPUS / WOS	26	European Journal of Sport Science	2,69	Q1
9	Arbulu et al.	SCOPUS / PSYCINFO / WOS	43	Cuadernos de Psicología del Deporte		
10	Arias-Pujol et al.	SCOPUS / WOS	10	Frontiers in Psychology	2,99	Q2
11	Argibay et al.	WOS		International Journal of Environmental Research and Public Health	4,614 (2021)	Q2
12	Asher et al.	WOS	56	Interface. Journal of the Royal Society	3,355 (2017)	Q1
13	Brill et al.	PSYCINFO	1	Frontiers in Psychology		
14	Brilot et al.	SCOPUS / PSYCINFO	21	Behavioural Processes	1,527	Q4
15	Burgoon et al.	OTHER SOURCES		Journal of Nonverbal Behavior	1,974	Q2
16	Camerino et al.	SCOPUS	5	Physiology and Behavior		
17	Camerino et al.	SCOPUS/ WOS	88	European Journal of Sport Science	1,146	Q3
18	Camerino et al.	SCOPUS / PSYCINFO / WOS	19	Revista de Psicología del Deporte	0,487	Q4
19	Camerino et al.	SCOPUS	29	Frontiers in Psychology		
20	Casarrubea et al.	PSYCINFO / SCOPUS	2	Brain Sciences		
21	Casarrubea et al.	OTHER SOURCES		Progress in Neuro-Psychopharmacology and Biological Psychiatry		
22	Casarrubea et al.	SCOPUS	3	Brain Sciences		
23	Casarrubea et al.	OTHER SOURCES		Neuroscience Letters		
24	Casarrubea et al.	OTHER SOURCES		Journal of Neuroscience Methods	2,785	Q2
25	Casarrubea et al.	OTHER SOURCES		Psychopharmacology	3,036	Q2
26	Castañer et al.	SCOPUS	7	Physiology & Behavior		
27	Castañer et al.	OTHER SOURCES		Frontiers in Psychology	2,129	Q2
28	Castañer et al.	SCOPUS / WOS	24	Procedia - Social and Behavioral Sciences		
29	Castañer et al.	OTHER SOURCES		Quality and Quantity	0,761	Q2
30	Castañer et al.	SCOPUS / PSYCINFO / WOS	3	Cuadernos de Psicología del Deporte		
31	Cavalera et al.	SCOPUS / PSYCINFO / WOS	12	Cuadernos de Psicología del Deporte		

32	Cenni et al.	PSYCINFO / SCOPUS	17	Physiology & Behavior		
33	Chaverri et al.	OTHER SOURCES		Gymnasium. Revista de Educação Física, Desporto e Saúde		
34	Conceição et al.	SCOPUS / WOS		Revista Brasileira de Cineantropometria e Desempenho Humano		
35	De Haas et al.	SCOPUS	23	Journal of Psychopharmacology	3,036	Q2
36	Diana et al.	SCOPUS / PSYCINFO / WOS	25	Frontiers in Psychology	2,089	Q2
37	Diana et al.	SCOPUS / PSYCINFO / WOS	4	Frontiers in psychology	2,129	Q2
38	Díaz-Aroca et al.	SCOPUS / PSYCINFO / WOS	1	Cuadernos de Psicología del Deporte		
39	Escolano-Pérez	SCOPUS		Sustainability	3,251	Q2
40	Escolano-Pérez et al.	OTHER SOURCES		Frontiers in Psychology	2,067	Q2
41	Fernández-Hermógenes et al.	SCOPUS	16	Apunts. Educación Física y Deportes		
42	Fernández-Hermógenes et al.	WOS		Apunts. Educación Física y Deportes		
43	García-Fariña et al.	SCOPUS / PSYCINFO / WOS	9	Cuadernos de Psicología del Deporte		
44	Garzón et al.	OTHER SOURCES		Psicothema	1,344	Q2
45	Garzón et al.	OTHER SOURCES		Revista de Psicología del Deporte	0,487	Q4
46	Garzón et al.	OTHER SOURCES		Revista Iberoamericana de Ciencias de la Actividad Física y el Deporte		
47	Gunst et al.	SCOPUS	6	Physiology and Behavior		
48	Gutiérrez-Santiago et al.	SCOPUS / PSYCINFO	6	Journal of Visual Impairment & Blindness	0,583	Q4
49	Gutiérrez-Santiago et al.	SCOPUS / PSYCINFO / WOS		Perceptual and Motor Skills	2,008 (2021)	Q3
50	Gutiérrez-Santiago et al.	PSYCINFO	2	International Journal of Performance Analysis in Sport		
51	Gutiérrez-Santiago et al.	PSYCINFO	1	Physiology and Behavior		
52	Gutiérrez-Santiago et al.	SCOPUS / PSYCINFO / WOS	16	International Journal of Sports Science & Coaching	0,815	Q3
53	Gutiérrez-Santiago et al.	SCOPUS / PSYCINFO / WOS	69	Journal of Sports Sciences	1,931	Q2
54	Gutiérrez-Santiago et al.	OTHER SOURCES		Apunts. Educación Física y Deportes		
55	Gutiérrez-Santiago et al.	SCOPUS / WOS	19	Journal of Sports Engineering and Technology	0,69	Q4
56	Gutiérrez-Santiago et al.	SCOPUS / WOS	12	Journal of Sports Science & Medicine	0,815	Q3
57	Gutiérrez-Santiago et al.	OTHER SOURCES		Revista Internacional de Medicina y Ciencias de la Actividad Física y del Deporte		
58	Hocking et al.	SCOPUS / PSYCINFO / WOS	21	Applied Animal Behaviour Science	1,823	Q1
59	Hunyadi	OTHER SOURCES		Frontiers in Psychology	2,067	Q2
60	Ibáñez et al.	SCOPUS / WOS	7	Ciencia, Cultura y Deporte		
61	Iglesias et al.	SCOPUS / PSYCINFO / WOS	4	Cuadernos de Psicología del Deporte		
62	Jonsson et al.	PSYCINFO	9	Behavior Research Methods		
63	Jonsson et al.	SCOPUS	26	The Open Sports Sciences Journal		
64	Kemp et al.	SCOPUS / WOS	45	Psychiatric Research	4,896	Q1
65	Kerepesi et al.	OTHER SOURCES		Behavioural Processes		
66	Kerepesi et al.	SCOPUS / PSYCINFO / WOS	73	Behavioural Processes	1,684	Q3

67	Lapresa et al.	SCOPUS / PSYCINFO / WOS	18	Revista de Psicología del Deporte	0,487	Q4
68	Lapresa et al.	SCOPUS / WOS	42	International Journal of Performance Analysis in Sport	0,741	Q3
69	Lapresa et al.	SCOPUS / WOS	88	Journal of Sports Sciences	2,246	Q1
70	Lapresa et al.	SCOPUS / WOS	3	Apunts. Educación Física y Deportes		
71	Lapresa et al.	SCOPUS / PSYCINFO / WOS	41	Cuadernos de Psicología del Deporte		
72	Lapresa et al.	OTHER SOURCES		Apunts. Educación Física y Deportes		
73	Lapresa et al.	SCOPUS / WOS	11	International Journal of Performance Analysis in Sport	1,325	Q4
74	Lapresa et al.	SCOPUS / WOS	8	Revista de Psicología del Deporte	0,543	Q4
75	Lapresa et al.	SCOPUS / WOS	19	International Journal of Performance Analysis in Sport	0,539 (2012)	Q4
76	Lapresa et al.	SCOPUS / PSYCINFO / WOS	4	Cuadernos de Psicología del Deporte		
77	Lapresa et al.	OTHER SOURCES		Journal of Development and Physical Disabilities		
78	Lapresa et al.	SCOPUS / WOS	2	Revista Iberoamericana de Psicología del Ejercicio y el Deporte		
79	Lavega et al.	SCOPUS / PSYCINFO / WOS	11	Frontiers in Psychology	2,99	Q2
80	Louro et al.	SCOPUS	16	Journal of Sports Science and Medicine	0,676	Q3
81	Lyon et al.	PSYCINFO / WOS	44	Psychopharmacology	3,146	Q1
82	Merlet et al.	SCOPUS / PSYCINFO / WOS	23	Applied Animal Behaviour Science	1,441	Q1
83	Pic	SCOPUS / WOS		Cuadernos de Psicología del Deporte		
84	Pic	WOS	4	E-Balonmano. Com: Revista de Ciencias del Deporte		
85	Pic	SCOPUS / WOS	7	Retos		
86	Pic	SCOPUS / WOS	25	Journal of Human Kinetics	1,414	Q3
87	Pic et al.	SCOPUS / PSYCINFO / WOS	30	Physiology and Behavior	3,244	Q2
88	Pic et al.	SCOPUS / PSYCINFO / WOS	18	Frontiers in Psychology	2,129	Q2
89	Pic et al.	SCOPUS / PSYCINFO / WOS	6	Physiology and Behavior	3,244	Q2
90	Portell et al.	OTHER SOURCES		Frontiers in Psychology	2,067	Q2
91	Prat et al.	SCOPUS	7	Frontiers in Psychology		
92	Prieto-Lage et al.	SCOPUS / PSYCINFO / WOS	1	Cuadernos de Psicología del Deporte		
93	Prieto-Lage et al.	PSYCINFO	5	International Journal of Sport Sciences & Coaching		
94	Prieto-Lage et al.	OTHER SOURCES		International Journal of Performance Analysis in Sport		
95	Prieto-Lage et al.	SCOPUS / WOS	3	Journal of Human Kinetics	1,029	Q3
96	Prieto-Lage et al.	SCOPUS / PSYCINFO / WOS	9	Physiology and Behavior	3,244	Q2
97	Prieto-Lage et al.	SCOPUS / PSYCINFO / WOS	10	Physiology and Behavior	3,244	Q2
98	Sandman et al.	SCOPUS / PSYCINFO / WOS	16	Journal of Intellectual Disability Research	1,81	Q1
99	Santangelo et al.	OTHER SOURCES	11	Physiology and Behavior		
100	Santos et al.	WOS	2	Revista Brasileira de Futsal e Futebol		
101	Santos et al.	SCOPUS / PSYCINFO / WOS	36	Motricidade		

102	Santos et al.	WOS		Frontiers in Psychology	4,232 (2021)	Q1
103	Santos et al.	SCOPUS / WOS		Motricidade		
104	Santoyo et al.	SCOPUS / PSYCINFO WOS	19	Anales de Psicología	0,8	Q4
105	Santoyo et al.	PSYCINFO	8	Physiology and Behavior	3,244	Q2
106	Sastre et al.	SCOPUS / WOS	5	International Journal of Performance Analysis in Sport	2,488	Q3
107	Sarmento et al.	OTHER SOURCES		Journal of Sports Sciences		
108	Sauch et al.	SCOPUS / WOS	11	Revista de Psicología del Deporte	0,487	Q4
109	Sene-Mir et al.	OTHER SOURCES		International Journal of Environmental Research and Public Health	3,39	Q1
110	Serna et al.	SCOPUS / WOS	9	Revista de Psicología del Deporte	0,922	Q4
111	Suárez et al.	SCOPUS / PSYCINFO / WOS	19	Frontiers in Psychology	2,129	Q2
112	Szekrényes	SCOPUS / PSYCINFO / WOS	22	Frontiers in Psychology	2,067	Q2
113	Tarragó et al.	SCOPUS / PSYCINFO / WOS	11	Cuadernos de Psicología del Deporte		
114	Tarragó et al.	OTHER SOURCES		Anales de Psicología	0,8	Q4
115	Tarragó et al.	SCOPUS / PSYCINFO / WOS	10	Cuadernos de Psicología del Deporte		
116	Terroba et al.	SCOPUS / WOS	2	Revista de Psicodidáctica	3,775	Q1
117	Torrents et al.	OTHER SOURCES		Education, Physical Training, Sport		
118	Torrents et al.	SCOPUS / PSYCINFO / WOS	40	Journal of Creative Behavior	1,156	Q3
119	Tripiana	SCOPUS / WOS	2	Estudios pedagógicos		
120	Tripiana et al.	WOS		Revista Internacional de Educación Musical	0,39	Q3
121	Valero et al.	OTHER SOURCES		International Journal of Environmental Research and Public Health		
122	Valero et al.	SCOPUS / WOS	12	Apunts. Educación Física y Deportes		
123	Wedl et al.	SCOPUS / PSYCINFO / WOS	42	Behavioural Processes	1,652	Q3
124	Zurloni et al.	SCOPUS / PSYCINFO / WOS	20	Revista de Psicología del Deporte	0,487	Q4
125	Zurloni et al.	SCOPUS / PSYCINFO / WOS	13	Psychology of Sport and Exercise	2,809	Q2
