# Supplementary material for: T-pattern detection in the scientific literature of this century: A systematic review
Source: Front Psychol. 2023 Mar 1;14:1085980. doi: 10.3389/fpsyg.2023.1085980 (PMC10015708; doi:10.3389/fpsyg.2023.1085980)
Supplement: Supplementary file 4 [file Table_4.pdf]

Table 4. Methodological characteristics of the primary documents [I, first part]

Code	Authors	Methodology	Design	Participants	Ethical standards	Instrument to collecting data	Number dimensions / categories
1	Alonso-Vega et al.	Observational		31-year-old client	Yes	Functional Coding System for Verbal Interaction in Clinical Contexts	5 clinician cat
2	Alsasua et al.	Observational	N/F/M	Men's teams from USA and Spain in the 2016 Paralympic Games	Yes	Ad hoc (adaptation of the SOBL-2)	7 dim, 48 cat
3	Alsasua et al.	Observational	N/F/M	ACB and U16 teams	Yes	Ad hoc (SOBL-2)	5 dim, 39 cat
4	Alves et al.	Observational	I/P/M	12 instructors		Ad hoc (SOCIN and SOPROX)	5 dim, 21 cat (SOCIN); 5 dim., 23 cat (SOPROX)
5	Alves et al.	Observational	N/P/M	12 instructors		Ad hoc (SOCIN-fitness and SOPROX-fitness)	5 dim, 21 cat (SOCIN); 5 dim., 23 cat (SOPROX)
6	Amatria et al.	Observational	N/F/M	20 players	Yes	Ad hoc (modified SOF)	7 dim, 49 cat
7	Amatria et al.	Observational	I/P/M	Spanish team UEFA	Yes	Ad hoc	
8	Aragón et al.	Observational	N/F/M	All finals on championships	Yes	Ad hoc	10 dim, 81 cat
9	Arbulu et al.	Observational	N/P/M	Final on World Championship		Ad hoc (SCOT)	6 dim, 74 cat
10	Arias-Pujol et al.	Observational	N/F/M	6 adolescents, 1 therapist, 1 co-therapist	Yes	Ad hoc	15 dim, 28 cat
11	Argibay et al.	Observational	N/F/U	277 injuries in 38 league games	Yes	Adaptation of OI-INJURIES-FOOTBALL	4 dim, 100 cat
12	Asher et al.						
13	Brill et al.	Experimental		61 participants (reduced to a 53)	Yes	Likert scale	5-point
14	Brilot et al.	Experimental		8 starlings (4 males + 4 females)	Yes	Ad hoc	15 cat
15	Burgoon et al.	Experimental		26 + 68 + 42 participants			
16	Camerino et al.	Observational	N/F/M	221 images from 6 magazines		Ad hoc (OSBI)	9 dim, 41 cat
17	Camerino et al.	Observational	N/P/M	10 competitions (5 National + 5 Champions)		Ad hoc (SOF5)	3 dim, 17 cat
18	Camerino et al.	Observational	N/P/M	50 participants	Yes	Ad hoc (SOBJUDO-KSGA)	9 dim, 33 cat
19	Camerino et al.	Observational		4 teachers and 120 students	Yes	Ad hoc (OSTOR) and TARE	6 dim. (OSTOR)
20	Casarrubea et al.	Experimental		20 adults (rats)	Yes	Ethogram	7 cat
21	Casarrubea et al.	Experimental		20 male rats	Yes	Ethogram	11 cat
22	Casarrubea et al.	Experimental		30 male rats		Ethogram	4 main cat and 11 behavioral components
23	Casarrubea et al.	Experimental		9 pairs of male rats	Yes	Ethograms (2)	10 cat and 11 cat
24	Casarrubea et al.	Observational and experimental					4 dim, 32 cat; 6 dim, 24 cat; 10 cat; 13 cat
25	Casarrubea et al.	Experimental		56 rats	Yes		11 cat
26	Castañer et al.	Observational	N/F/M	68 non-professional athletes	Yes	Ad hoc (OSMOSTI)	8 dim, 26 cat
27	Castañer et al.	Observational	N/F/M	2 participants (181 goals)		Ad hoc (OSMOS-soccer)	9 dim, 50 cat
28	Castañer et al.	Observational		4 novice teachers + 4 expert teachers		Ad hoc (SOCIN, and SOPROX)	3 dim, 17 cat (SOCIN); 5 dim, 17 cat (SOPROX)
29	Castañer et al.	Observational		4 novice teachers + 4 expert teachers	Yes	Ad hoc (SOCIN, and SOPROX)	3 dim, 17 cat (SOCIN); 5 dim, 17 cat (SOPROX)
30	Castañer et al.	Mixed methods	Embedded	90 participants	Yes	Ad hoc (OSMOS-in context) and Questionnaire	10 dim, 31 cat
31	Cavalera et al.	Observational	N/P/M	19 matches		Ad hoc	6 dim

32	Cenni et al.	Observational		6 and 14 (from 700 individuals)	Yes		
33	Chaverri et al.	Observational	N/P/M	All matches FCB (2020-2021)		Ad hoc (SOF5)	5 dim var, 31 cat
34	Conceição et al.	Experimental		5 swimmers (national level)	Yes	EMG equipment	5 dim
35	De Haas et al.	Experimental			Yes		
36	Diana et al.	Observational	I/P/M	All matches Italian League (2012-2013)	Yes	Ad hoc	6 dim, 29 cat
37	Diana et al.	Experimental		46 students			7 dim
38	Díaz-Aroca et al.	Observational	N/P/M	12 players	Yes	Ad hoc	10 dim, 22 cat (prior); 9 dim, 25 cat (ball rel)
39	Escolano-Pérez	Observational	N/F/M	32 twin todlers	Yes	Ad hoc	4 dim, 29 cat
40	Escolano-Pérez et al.	Observational	N/P/M	44 children	Yes	Ad hoc	5 dim, 21 cat
41	Fernández-Hermógenes et al.	Observational	N/P/M	52 matchs		Ad hoc (SOFEO)	6 dim, 34 cat
42	Fernández-Hermógenes et al.	Observational	I-N/P/M	20+22 soccer teams		Ad hoc (SOCFO-1)	11 dim, 47 cat
43	García-Fariña et al.	Observational	I/F/M	1 teacher + 19 students	Yes	Ad hoc (ADDEF)	9 dim, 32 cat
44	Garzón et al.	Observational	N/P/M	9 players		Ad hoc	15 dim, 64 cat (var)
45	Garzón et al.	Observational	N/P/M	9 players		Ad hoc	19 dim, 71 cat
46	Garzón et al.	Observational and quasiexperimental	N/P/M	9 players		Ad hoc	19 dim, 71 cat
47	Gunst et al.	Observational		98 (females) + 18 (males). 56 focal subjects	Yes	Ethogram	
48	Gutiérrez-Santiago et al.	Observational		184 combats and 92 male senior judokas	Yes	Ad hoc (OTSJUDO)	2 dim, 11 cat
49	Gutiérrez-Santiago et al.	Observational and quasiexperimental	N/P/M	54 adolescents	Yes	Ad hoc (IOUPPERLIMB_FLEX_EXT)	12 dim, 21 cat
50	Gutiérrez-Santiago et al.	Observational	N/F/M	76 combats	Yes	Ad hoc (SA-JUDO)	9 dim, 49 cat; 4 dim, 11 cat
51	Gutiérrez-Santiago et al.	Observational	N/F/M	35 combats	Yes	Ad hoc (SA-TAEKWONDO)	10 dim, 51 cat
52	Gutiérrez-Santiago et al.	Observational		92 senior judokas	Yes	Ad hoc (OTSJUDO)	2 dim, 11 cat
53	Gutiérrez-Santiago et al.	Observational	N/M/M	117 senior judokas	Yes	Ad hoc (OTSJUDO)	2 dim, 11 cat
54	Gutiérrez-Santiago et al.	Observational	N/M/M	117 senior judokas	Yes	Ad hoc (OTSJUDO)	16 dim, 39 cat
55	Gutiérrez-Santiago et al.	Observational		46 judokas		Ad hoc (OTSJUDO)	13 dim, 21 cat
56	Gutiérrez-Santiago et al.	Observational	N/F/M	46 judokas	Yes	Ad hoc (OTSJUDO)	13 dim, 60 cat
57	Gutiérrez-Santiago et al.	Observational	N/F/M	46 judokas		Ad hoc (OTSJUDO)	12 dim, 19 cat
58	Hocking et al.	Experimental	2x2 randomised block factorial	408 standard broiler breeder female chicks			16 cat
59	Hunyadi	Experimental					9 cat
60	Ibáñez et al.	Observational	N/F/M	16 contestants	Yes	Ad hoc (OBKA)	8 dim, 53 cat
61	Iglesias et al.	Observational	N/P/M	26 female swimmers	Yes	Ad hoc (SINCROBS)	4 dim, 13 cat
62	Jonsson et al.	Observational	N/P/M	6 matches		Ad-hoc (SOF-CODER)	5 dim, 30 cat
63	Jonsson et al.	Observational					
64	Kemp et al.	Observational		25 participants	Yes		
65	Kerepesi et al.	Observational		7 owners			21 behavior unis
66	Kerepesi et al.	Observational and experimental		28 adults and 28 children			3 dim, 25 cat

67	Lapresa et al.	Observational	I/P/M	RM Matches 2012		Ad hoc (SOBL-2)	5 dim, 38 cat
68	Lapresa et al.	Observational	I/P/M	8 children's teams	Yes	Ad hoc (SOBL-2)	5 dim, 38 cat
69	Lapresa et al.	Observational	N/P/M	3 teams	Yes	Ad hoc	8 dim, 65 cat
70	Lapresa et al.	Observational	I/F/M	Sergio Busquets player		Ad hoc	13 dim, 100 cat
71	Lapresa et al.	Observational	N/F/M	7 matchs		Ad hoc (SOFS)	8 dim, 41 cat
72	Lapresa et al.	Observational	N/P/M	Goalkeepers of tournament		Ad hoc	8 dim, 47 cat
73	Lapresa et al.	Observational	I/F/M	12 teams		Ad hoc (adapted from SOF-4 and SOFBAS)	6 dim, 45 cat
74	Lapresa et al.	Observational and quasiexperimental		Tournement		Ad hoc	9 dim
75	Lapresa et al.	Observational	N/P/M	11+18 combats	Yes	Ad hoc	11 dim, 57 cat
76	Lapresa et al.	Observational	N/F/M	6 players		Ad hoc	5 dim, 29 cat
77	Lapresa et al.	Observational	N/F/M	4 players		Ad hoc	12 dim; 119 cat (var)
78	Lapresa et al.	Observational	N/F/M	5 athlets		Ad hoc	14 dim, 49 cat
79	Lavega et al.	Observational and quasiexperimental	N/P/M	14 players	Yes	Ad hoc	6 dim, 46 cat
80	Louro et al.	Observational	N/P/M	4 international level swimmers		Ad hoc (adapted from SOCTM)	4 dim
81	Lyon et al.	Experimental		62 psychiatric patients and 33 normal subjects	Yes	Two-choice (rigth/left) task	2 cat
82	Merlet et al.	Observational and experimental		672-day-olf female chicks		Ad hoc ethogram	4 dim, 29 cat
83	Pic	Observational	N/P/M	2 players		Ad hoc	4 dim, 11 cat
84	Pic	Observational	N/P/M	2 men's matches, 2 women's matches		Ad hoc	5 dim, 15 cat
85	Pic	Observational		38+38 matches		Ad hoc	7 dim, 17 cat
86	Pic	Observational	N/P/M	39 matches		Ad hoc	7 dim, 36 cat
87	Pic et al.	Observational	N/P/M	15 boxing figths		Ad hoc	5 dim, 21 cat
88	Pic et al.	Observational	N/P/M	84 players		Ad hoc	4 dim, 15 cat
89	Pic et al.	Observational	N/P/M	23 players		Ad hoc	4 dim, 17 cat
90	Portell et al.	Observational	N/F/M	53 blue-collar workers	Yes	Ad hoc (SsObserWork)	6 dim, 21 cat (H-O); 5 dim, 15 cat (S-O)
91	Prat et al.	Mixed methods	Embedded design	71 participants	Yes	Ad hoc (OSMOS, MOTORLAT, PATHoops)	8 dim, 25 cat; 4 dim, 30 items; 2 dim, 14 cat
92	Prieto-Lage et al.	Observational	N/P/M	121 penalties		Ad hoc (PENALTY KICK PLAYER)	11 dim, 41 cat
93	Prieto-Lage et al.	Observational	N/M/M	55 students	Yes	Ad hoc (SOBJUDO-OSG)	8 dim, 20 cat
94	Prieto-Lage et al.	Observational		193 novice students	Yes	Ad hoc (OBSJUDO)	13 dim
95	Prieto-Lage et al.	Observational	N/M/M	45 students	Yes	Ad hoc (SOBJUDO-OSGU)	10 dim, 21 cat
96	Prieto-Lage et al.	Observational	N/F/U	116 injuries	Yes	Ad hoc	16 dim, 90 cat
97	Prieto-Lage et al.	Observational	N/P/M	78 students	Yes	Ad hoc (OI-JUGO-TG)	12 dim, 45 cat
98	Sandman et al.	Observational		18+14 participants	Yes		6 cat
99	Santangelo et al.			12 individuals		Ethogram (TSST)	5 cat (non-verbal); 4 cat (verbal)
100	Santos et al.	Observational		65 goals		Ad hoc (SOGF)	4 dim, 48 cat
101	Santos et al.	Observational	N/P/M	4 coaches		Ad hoc (SAIC)	4 dim, 22 cat, 26 subcat

102	Santos et al.	Observational	I/F/M	3 U-17 GKs, 30 football matches	Yes	Ad hoc	4 dim, 34 cat
103	Santos et al.	Observational	I/P/U	3 coaches	Yes	Ad hoc (SAIPCTA)	11 dim, 35 cat; 2 cat; 3 dim, 14 cat; 3 cat
104	Santoyo et al.	Observational	I/F/M	28 students	Yes	Ad hoc (SOC-IS)	5 dim, 13 cat
105	Santoyo et al.	Observational	N/F/M	13+17 children		Ad hoc (OBBSI)	4 dim, 105 cat
106	Sastre et al.	Observational	N/F/M	37 participants	Yes	Ad hoc (OBKA)	7 dim, 38 cat
107	Sarmento et al.	Observational		30 games (126 goals scored)	Yes	Ad hoc	6 dim, 69 cat
108	Sauch et al.	Mixed methods	Multilevel triangulation	50 users	Yes	Ad hoc (OSMOS)	11 dim, 34 cat
109	Sene-Mir et al.	Observational and experimental	N/F/M	31+30 blue-collar workers	Yes	Ad hoc (SsObserWork)	6 dim
110	Serna et al.	Observational	I/F/M	14 matches		Ad hoc (SOCCB)	6 dim, 27 cat
111	Suárez et al.	Observational	N/F/M	6 teachers		Ad hoc	14 dim, 72 cat
112	Szekrényes	Experimental					4 dim
113	Tarragó et al.	Observational	N/P/M	8 assaults by 2 fencers	Yes	Ad hoc (ESGRIMOBS)	6 dim, 25 cat
114	Tarragó et al.	Observational	N/P/M	24 bouts	Yes	Ad hoc (ESGRIMOBS)	10 dim, 51 cat
115	Tarragó et al.	Observational	N/P/M	16 fencers	Yes	Ad hoc (ESGRIMOBS)	6 dim, 26 cat
116	Terroba et al.	Observational	N/F/M	24 students	Yes	Ad hoc	11 dim, 68 cat
117	Torrents et al.	Observational	N/P/M	7 students		Ad hoc	4 dim, 15 cat
118	Torrents et al.	Observational		4 dancers		Ad hoc (OSMOS)	6 dim, 27 cat
119	Tripiana	Observational	I/F/M	30 students		Ad hoc	9 dim (music); 6 dim (interpretation)
120	Tripiana et al.	Observational	I/F/M	30 students		Ad hoc	9 dim (music); 6 dim (interpretation)
121	Valero et al.	Mixed methods	Multilevel triangulation	44 sessions	Yes	OSTOR (SORPS), EME, PSRQ, PNSE, CECSE, CUVE	OSTOR: 6 dim, 18 cat
122	Valero et al.	Mixed methods	Multilevel triangulation	28+27 students	Yes	Ad hoc (SORPS) + questionnaire	6 dim, 22 cat
123	Wedl et al.	Observational and experimental		40 cats and 39 owners		Ad hoc + interview	
124	Zurloni et al.	Observational	N/P/M	12 games		Ad hoc	6 dim, 29 cat
125	Zurloni et al.	Observational and interviews		1 professional of cycling		FACS	
