# Supplementary material for: T-pattern detection in the scientific literature of this century: A systematic review
Source: Front Psychol. 2023 Mar 1;14:1085980. doi: 10.3389/fpsyg.2023.1085980 (PMC10015708; doi:10.3389/fpsyg.2023.1085980)
Supplement: Supplementary file 5 [file Table_5.pdf]

Table 5. Methodological characteristics of the primary documents [I, second part]

Code	Authors	Software record	Software control quality of data	Control quality of data	Software analysis	Data analysis
1	Alonso-Vega et al.	Observer	GSEQ	Kappa	THEME and GSEQ5	T-Patterns, lag sequential analysis, Yules'Q, and contingency index
2	Alsasua et al.	LINCE	LINCE	Kappa	THEME	T-Patterns
3	Alsasua et al.	MATCH VISION STUDIO	GSEQ	Kappa and TG	THEME 5.0 and SPSS	T-Patterns and χ^2
4	Alves et al.	LINCE	LINCE	Kappa	THEME 5.0	T-Patterns
5	Alves et al.	LINCE	LINCE	Kappa	THEME 5.0 and SPSS	T-Patterns and descriptive analysis
6	Amatria et al.	LINCE	LINCE	Kappa	THEME 5.0	T-Patterns
7	Amatria et al.	LINCE	LINCE and SAGT	Kappa and TG	THEME Edu	T-Patterns
8	Aragón et al.	MATCH VISION STUDIO	GSEQ	Kappa and TG	THEME 5.0 and SPSS	T-Patterns and χ^2
9	Arbulu et al.	MOTS		Kappa and TG	THEME 6.0 and SPSS	T-Patterns
10	Arias-Pujol et al.	GSEQ	GSEQ	Kappa	THEME Edu, GSEQ, and HOISAN	T-Patterns, lag sequencial, and polar coordinate
11	Argibay et al.	Excel	LINCE	Kappa	THEME 5.0	T-Patterns and χ^2
12	Asher et al.				THEME, GSEQ	T-Patterns, fractal, social network, agent-based and simulation
13	Brill et al.	Observer XT	Observer XT	Kappa	THEME	T-Patterns and group comparisons
14	Brilot et al.	J-Watcher			THEME, SPSS and SAS	T-Patterns and Markov chains
15	Burgoon et al.	Observer and C-BAS			THEME	T-Patterns
16	Camerino et al.	LINCE	LINCE	Kappa	THEME	T-Patterns
17	Camerino et al.	MATCH VISION STUDIO		Kappa	THEME	T-Patterns
18	Camerino et al.	LINCE	LINCE	Kappa	THEME and SPSS	T-Patterns and χ^2
19	Camerino et al.	LINCE and LINCE PLUS	LINCE	Kappa	THEME and HOISAN	T-Patterns, polar coordinate analysis, and descriptive analysis
20	Casarrubea et al.	THE OBSERVER			THEME	T-Patterns and Fisher test
21	Casarrubea et al.	THE OBSERVER			THEME	T-Patterns and analysis of variance
22	Casarrubea et al.	THE OBSERVER			THEME	T-Patterns, analysis of variance, χ^2, and Newman-Keuls post-hoc test
23	Casarrubea et al.	THE OBSERVER			THEME	T-Patterns
24	Casarrubea et al.				THEME Edu	T-Patterns
25	Casarrubea et al.	Observer			THEME	T-Patterns
26	Castañer et al.	LINCE and LINCE PLUS	LINCE PLUS	Kappa	THEME	T-Patterns
27	Castañer et al.	LINCE	LINCE	Kappa	THEME Edu and HOISAN	T-Patterns and polar coordinate analysis
28	Castañer et al.	SDIS-GSEQ			THEME and SDIS-GSEQ	T-Patterns and lag sequential analysis
29	Castañer et al.	SDIS-GSEQ	SDIS-GSEQ	Kappa	THEME and SDIS-GSEQ	T-Patterns and lag sequential analysis
30	Castañer et al.	LINCE	LINCE	Kappa	THEME 6.0	T-Patterns

31	Cavalera et al.	LINCE	LINCE	Kappa	THEME 6.0	T-Patterns
32	Cenni et al.	THE OBSERVER			THEME	T-Patterns, Wilcoxon test, and t Student
33	Chaverri et al.	MATCH VISION STUDIO		Kappa	THEME	T-Patterns
34	Conceição et al.				THEME 5.0	T-Patterns
35	De Haas et al.	ETHOVISION			THEME	T-Patterns
36	Diana et al.	LINCE		Kappa	THEME 6.0	T-Patterns and Mann-Whitney test
37	Diana et al.	OBSERVER and RESEARCH RANDOMIZER		Kappa	Theme 6.0 and SPSS	T-Patterns and descriptive analysis
38	Díaz-Aroca et al.	Virtual Dub + Excel	SAGT	Kappa, Rho, Tau, %A, and TG	THEME Edu	T-Patterns
39	Escolano-Pérez	MATCH VISION STUDIO	SAS and EduG	TG	Theme 6.0	T-Patterns
40	Escolano-Pérez et al.	LINCE	SAS	TG	THEME 6.0, HOISAN and GSEQ	T-Patterns, polar coodinate and lag sequential analysis
41	Fernández-Hermógenes et al.	LINCE	LINCE	Kappa	THEME, SPSS	T-Patterns, Mann-Whitney test and χ^2
42	Fernández-Hermógenes et al.	LINCE PLUS	LINCE PLUS	Kappa	THEME and Stata/IC v. 15.1	T-Patterns and descriptive analysis
43	García-Fariña et al.	ATLAS.ti	HOISAN	Kappa & Canonical concordance	THEME 6.0	T-Patterns
44	Garzón et al.	THEMECODER	GSEQ	Kappa	THEME	T-Patterns
45	Garzón et al.	THEMECODER		Kappa	THEME and SPSS	T-Patterns and χ^2
46	Garzón et al.	THEMECODER	SDIS-GSEQ	Kappa and TG	THEME and SPSS	T-Patterns and χ^2
47	Gunst et al.	OBSERVER			THEME	T-Patterns, t Student, Mann-Whitney, and Kruskal-Wallis tests
48	Gutiérrez-Santiago et al.	MATCH VISION STUDIO	GSEQ	Kappa	THEME	T-Patterns
49	Gutiérrez-Santiago et al.	LINCE	LINCE	Kappa	THEME, HOISAN and SPSS	T-Patterns, polar coordinate and ANOVA
50	Gutiérrez-Santiago et al.	LINCE	LINCE	Kappa	THEME 5.0, GSEQ, HOISAN, and SPSS	T-Patterns, lag sequential analysis, and polar coordinate analysis
51	Gutiérrez-Santiago et al.	LINCE and LINCE PLUS	LINCE	Kappa	THEME 5.0, GSEQ, HOISAN, and SPSS	T-Patterns, lag sequential analysis, and polar coordinate analysis
52	Gutiérrez-Santiago et al.	MATCH VISION STUDIO	GSEQ	Kappa	THEME and SPSS	T-Patterns, ANOVA, and χ^2
53	Gutiérrez-Santiago et al.	MATCH VISION STUDIO	GSEQ	Kappa	THEME 5.0	T-Patterns
54	Gutiérrez-Santiago et al.	MATCH VISION STUDIO	SDIS-GSEQ	Kappa	THEME and SPSS	T-Patterns and χ^2
55	Gutiérrez-Santiago et al.	MATCH VISION STUDIO	SDIS-GSEQ	Kappa	THEME	T-Patterns
56	Gutiérrez-Santiago et al.	MATCH VISION STUDIO	SDIS-GSEQ	Kappa	THEME and SPSS	T-Patterns and descriptive analysis
57	Gutiérrez-Santiago et al.	MATCH VISION STUDIO	GSEQ	Kappa	THEME and SPSS	T-Patterns and Mann-Whitney test
58	Hocking et al.				THEME	T-Patterns, ANOVA, and fractals
59	Hunyadi				THEME	T-Patterns
60	Ibáñez et al.	THEMECODER		Kappa and TG	THEME and SPSS	T-Patterns and χ^2
61	Iglesias et al.	LINCE	LINCE	Consensus agreement and Kappa	THEME	T-Patterns
62	Jonsson et al.	SOF-CODER		Kappa	THEME	T-Patterns
63	Jonsson et al.				THEME	T-Patterns
64	Kemp et al.		SPSS	Pearson correlation	THEME and SPSS	T-Patterns and Pearson correlation
65	Kerepesi et al.				THEME	T-Patterns and ANOVA

66	Kerepesi et al.	THEMECODER			THEME 5.0	T-Patterns and ANOVA
67	Lapresa et al.	MATCH VISION STUDIO	GT and SPSS	TG	THEME	T-Patterns
68	Lapresa et al.	MATCH VISION STUDIO	SPSS	TG	THEME 5.0	T-Patterns
69	Lapresa et al.	SDIS-GSEQ	SDIS-GSEQ	Kappa	THEME and SDIS-GSEQ	T-Patterns and lag sequential analysis
70	Lapresa et al.	LINCE	SDIS-GSEQ	Kappa and TG	THEME and GSEQ5	T-Patterns and lag sequential analysis
71	Lapresa et al.	LINCE	LINCE	Kappa	THEME 6.0	T-Patterns
72	Lapresa et al.	LINCE	GSEQ	Kappa	THEME	T-Patterns and χ^2
73	Lapresa et al.	LINCE	GSEQ and SAGT	Kappa and TG	THEME and GSEQ5	T-Patterns and lag sequential analysis
74	Lapresa et al.	THEMECODER	SDIS-GSEQ	Kappa	THEME 5.0	T-Patterns
75	Lapresa et al.	THEMECODER	SDIS-GSEQ and SPSS	Kappa and TG	THEME and SPSS	T-Patterns and comparative analysis
76	Lapresa et al.	LINCE	GSEQ and SAGT	Kappa and TG	THEME Edu	T-Patterns
77	Lapresa et al.	LINCE		Kappa	THEME	T-Patterns
78	Lapresa et al.	MATCH VISION STUDIO	GSEQ and SAGT	Kappa and TG	THEME 5.0	T-Patterns
79	Lavega et al.	Excel		Consensus agreement and TG	THEME	T-Patterns
80	Louro et al.				THEME	T-Patterns and descriptive analysis
81	Lyon et al.				THEME and SAS	T-Patterns and GLM analysis of variance
82	Merlet et al.	OBSERVER			THEME	T-Patterns, transition diagrams, correlation, and ANOVA
83	Pic	LINCE		Pearson coefficient and TG	THEME and SPSS	T-Patterns and multivariate analysis
84	Pic	LINCE	SPSS	Pearson and Spearman correlation	THEME 6.0	T-Patterns
85	Pic				THEME	T-Patterns
86	Pic	LINCE		Pearson and Spearman coefficients	THEME and SPSS	T-Patterns and decision trees
87	Pic et al.	LINCE		Kendall and Spearman coefficients	THEME	T-Patterns and χ^2
88	Pic et al.	LINCE	SAS, SPSS, and GT	Pearson and Spearman coefficients and TG	THEME	T-Patterns
89	Pic et al.	LINCE	SAS, SPSS, and GT	Pearson and Spearman coefficients and TG	THEME	T-Patterns and χ^2
90	Portell et al.	LINCE	GSEQ	Kappa	THEME and HOISAN	T-Patterns and polar ccordinate analysis
91	Prat et al.	LINCE			THEME	T-Patterns
92	Prieto-Lage et al.	LINCE	LINCE	Kappa	THEME, GSEQ and HOISAN	T-Patterns, lag sequential and polar coordinate analysis
93	Prieto-Lage et al.	MATCH VISION STUDIO	GSEQ	Kappa	THEME and SPSS	T-Patterns and descriptive analysis
94	Prieto-Lage et al.	LINCE	GSEQ	Kappa	THEME 5.0 and SPSS	T-Patterns
95	Prieto-Lage et al.	MATCH VISION STUDIO	GSEQ	Kappa	THEME 5.0	T-Patterns
96	Prieto-Lage et al.	LINCE		Kappa	THEME, GSEQ, HOISAN and SPSS	T-Patterns, lag sequential, polar coordinate analysis and χ^2
97	Prieto-Lage et al.	LINCE		Kappa	THEME, GSEQ, HOISAN and SPSS	T-Patterns, lag sequential, polar coordinate analysis and χ^2
98	Sandman et al.	THE OBSERVER		Pearson coefficient	THEME	T-Patterns
99	Santangelo et al.				THEME	T-Patterns, ANOVA, and Bonferroni test
100	Santos et al.	LINCE		Kappa	THEME, GSEQ5 and SPSS	T-Patterns, lag sequencial and descriptive analysis

101	Santos et al.	LINCE		Kappa	THEME 5.0	T-Patterns
102	Santos et al.	LINCE		Kappa	THEME 5.0	T-Patterns
103	Santos et al.	LINCE		Kappa and TG	THEME 6.0 and SPSS	T-Patterns and descriptive analysis
104	Santoyo et al.	SDIS-GSEQ	SDIS-GSEQ	Kappa	THEME, SDIS-GSEQ, HOISAN and STATGRAPHICS	T-Patterns, lag sequential, polar coordinate and trend analysis
105	Santoyo et al.	SDIS-GSEQ	GSEQ	Kappa and TG	THEME 6.0	T-Patterns
106	Sastre et al.	LINCE	GSEQ	Kappa and TG	THEME and GSEQ5	T-Patterns and lag sequential analysis
107	Sarmento et al.				THEME 5.0 and SPSS	T-Patterns and descriptive statistics
108	Sauch et al.	LINCE	SDIS-GSEQ	Kappa	THEME, NVIVO and SPSS	T-Patterns, content and descriptive analysis
109	Sene-Mir et al.	EXCEL		Kappa	THEME and R	T-Patterns, Shapiro-Wilk and Mann-Witney test
110	Serna et al.	LINCE	SPSS and SAGT	Kappa and TG	THEME and SPSS	T-Patterns and χ^2
111	Suárez et al.	MATCH VISION STUDIO	GT and SAS	TG	THEME	T-Patterns
112	Szekrényes				THEME	T-Patterns
113	Tarragó et al.	LINCE	GSEQ	Kappa	THEME and HOISAN	T-Patterns and polar coordinate analysis
114	Tarragó et al.	LINCE	GSEQ	Canonical agreement and Kappa	THEME, GSEQ and HOISAN	T-Patterns, lag sequential and polar coordinate analysis
115	Tarragó et al.	LINCE	GSEQ	Consensus agreement and kappa	THEME and SPSS	T-Patterns and ANOVA
116	Terroba et al.	LINCE	LINCE and SAGT	Kappa and TG	THEME	T-Patterns
117	Torrents et al.	MATCH VISION STUDIO		Kappa	THEME	T-Patterns
118	Torrents et al.	THEMECODER			THEME	T-Patterns
119	Tripiana	THEMECODER	GSEQ	Kappa	THEME	T-Patterns
120	Tripiana et al.	THEMECODER	GSEQ	Kappa	THEME	T-Patterns
121	Valero et al.	LINCE PLUS	LINCE PLUS	Kappa	THEME and SPSS	T-Patterns, Wilcoxon and Kolmogorov-Smirnov tests
122	Valero et al.	LINCE		Kappa	THEME and SPSS	T-Patterns and descriptive and inferential analysis
123	Wedl et al.	THE OBSERVER			THEME and SPSS	T-Patterns and general lineal model
124	Zurloni et al.	LINCE	LINCE	Kappa	THEME	T-Patterns
125	Zurloni et al.			Kappa	THEME	T-Patterns
