# Supplementary material for: T-pattern detection in the scientific literature of this century: A systematic review
Source: Front Psychol. 2023 Mar 1;14:1085980. doi: 10.3389/fpsyg.2023.1085980 (PMC10015708; doi:10.3389/fpsyg.2023.1085980)
Supplement: Supplementary file 6 [file Table_6.pdf]

Table 6. Methodological characteristics of the primary documents [II]

Code	Authors	Minimum occurrence	Level of significance	Redundancy reduction	Deact of fast req.	Criteria selection T-Patterns	Validation of results: Randomization	Results presentation	Selected T-Patterns
1	Alonso-Vega et al.							Tables	17 (2 terms) + 7 (3 terms) +3 (4 terms)
2	Alsasua et al.	5	0.005			Qualitative filters		Tables	16 effect sequences, 20 ineffct sequences
3	Alsasua et al.	3	0.005			Simulation filters	Randomization	Tables	7 (U16), 9 (ACB)
4	Alves et al.	3	0.05		Yes		Randomization	Tables & figures (with pictures)	456+458 (selected 5 + 5)
5	Alves et al.	3	0.05				Randomization	Tables & figures (with pictures)	127+229+358+130 (selected 5+5+5+5)
6	Amatria et al.	3	0.005	90%	Yes	Simulation filters; qualit & quant filters	Randomization	Tables	F7: 145+38+1; F8:160+42+1
7	Amatria et al.	2	0.05	90%	Yes	Simulation filters; qualit & quant filters	Randomization	Tables & figures (with pictures)	987 (2 cl) + 387 (3 cl) + 72 (4 cl) + 16 (5 cl) + 3 (6 cl)
8	Aragón et al.	2	0.05	90%	Yes	In function of critical interval		Tables	7 (1500 m), 8 (5000 m)
9	Arbulu et al.	3	0.005		Yes		Rand. (Shuf. & rot.)		3 (4, 3, and 2 occurrences)
10	Arias-Pujol et al.	30	0.005		Not			Tables & figures	4 (initial session), 2 (final session)
11	Argibay et al.	5	0.005	90%				Tables	Detected: 12686 (La Liga), 10818 (Premier)
12	Asher et al.								
13	Brill et al.	3	0.005			Qualitative filters (blinks)	Rand. (Shuf.& rot.)	Tables & Figures	43 and 42 (optical flow); 139 and 134 (cinematic structure)
14	Brilot et al.	Medium	0.001					Tables & figures	
15	Burgoon et al.			88,70%				Tables & figures	From 7 to 4.444 (decept), from 96 to 4.383 (truth)
16	Camerino et al.		0.005	90,00%		Quantitative filter (25 %)		Tables & Figures	
17	Camerino et al.	3	0.005					Tables & figures	66, 57, 46, 39, 22, 69, 23, 66, 51, 68
18	Camerino et al.	3	0.05		Yes			Tables & figures (with pictures)	
19	Camerino et al.							Tables & figures	1 of each teacher (21 branches)
20	Casarrubea et al.							Tables & figures	18
21	Casarrubea et al.	6	0.0001	90%				Tables & figures	17, 17, 41, 33
22	Casarrubea et al.	6	0.0001	90%				Tables & figures	48, 96, 18, 14
23	Casarrubea et al.	9	0.0001	90%				Figures	71, 65, 50, 27, 8
24	Casarrubea et al.								
25	Casarrubea et al.	8	0.0001				Randomization	Tables & figures	2750, 2289, 1937, 1762
26	Castañer et al.								
27	Castañer et al.	2	0.005	90%				Tables & figures	First 25 t-Patterns (more than 2 oc)
28	Castañer et al.							Figures	3 (novice teacher); 3 (expert teacher)
29	Castañer et al.							Figures	3 (novice teacher); 3 (expert teacher)
30	Castañer et al.							Figures	5

31	Cavalera et al.	3	0.005					Tables & figures	9, 30 (first); 51,85 (second); 21, 52 (third)
32	Cenni et al.						Randomization	Figures	75, 21
33	Chaverri et al.	3	0.005					Tables & figures	68
34	Conceição et al.								44
35	De Haas et al.	3	0.0001					Tables & figures	
36	Diana et al.		0.005					Tables & figures	82 (home); 20 (away)
37	Diana et al.	2	0.005					Tables & figures	
38	Díaz-Aroca et al.	3	0.005	90%			Randomization	Tables	
39	Escolano-Pérez	3	0.005	90%		Qualitative filters	Randomization	Tables	H: 4,5,3 (T1), 0,1,3 (T2), 1,1,3 (T3); L: 4,14,17 (T1), 2,4,15 (T2), 3,2,19 (T3)
40	Escolano-Pérez et al.	7	0.005		Yes	Qualitative filters	Randomization		11
41	Fernández-Hermógenes et al.		0.05					Tables & figures (with pictures)	
42	Fernández-Hermógenes et al.	3	0.005	90%				Tables & figures (with pictures)	
43	García-Fariña et al.							Figures	1 (before); 1 (after)
44	Garzón et al.	3	0.005				Randomization		
45	Garzón et al.		0.005			Simulation filters	Randomization	Tables & figures (with pictures)	
46	Garzón et al.		0.005				Randomization	Tables & figures (with pictures)	
47	Gunst et al.							Figures	20, 20
48	Gutiérrez-Santiago et al.							Tables & figures	
49	Gutiérrez-Santiago et al.	3	0.005					Tables & figures	25 (the most relevants)
50	Gutiérrez-Santiago et al.							Tables & figures	
51	Gutiérrez-Santiago et al.							Figures	481 (considered only 149)
52	Gutiérrez-Santiago et al.							Tables & figures	
53	Gutiérrez-Santiago et al.							Tables & figures	
54	Gutiérrez-Santiago et al.							Figures (with pictures)	
55	Gutiérrez-Santiago et al.							Figures (with pictures)	
56	Gutiérrez-Santiago et al.							Figures	
57	Gutiérrez-Santiago et al.							Figures (with pictures)	
58	Hocking et al.	3	0.0005					Tables	
59	Hunyadi	3	0.005					Tables & figures	10 (2 ev); 10 (3 ev); 10 (4 ev); 10 (5 ev); 5 (6 ev)
60	Ibáñez et al.	5 and 4	0.005	90%	Yes		Randomization	Tables & figures (with pictures)	35
61	Iglesias et al.	20	0.005					Tables & figures	23
62	Jonsson et al.	3	0.005					Figures	21
63	Jonsson et al.							Tables & figures (with pictures)	
64	Kemp et al.	3	0.000001					Tables & figures	
65	Kerepesi et al.						Randomization	Tables & figures	218 (total) and 181 interactive

66	Kerepesi et al.	2	0.005					Figures	
67	Lapresa et al.	2	0.005			Simulation filter	Randomization	Tables & figures	6; 4; 5
68	Lapresa et al.	3	0.005				Randomization	Tables & figures	17; 13; 18
69	Lapresa et al.	3	0.005		Yes	Simulation filter	Randomization	Tables & figures	10 (7-S); 11 (9-S); 6 (11-S)
70	Lapresa et al.	3	0.005	99%				Tables & figures	5
71	Lapresa et al.	3	0.005	Activated	Yes			Tables & figures	19 (lev 1); 12 (lev 2); 5 (lev 3); 2 (lev 4)
72	Lapresa et al.	3	0.005				Randomization	Tables & figures	30 (DTA); 30 (OTA)
73	Lapresa et al.	2	0.005	90%	Yes	Qualitative filters	Rand. (Shuf. & rot.)	Tables	6
74	Lapresa et al.	3	0.005		Yes	Simulation filter	Randomization	Figures	
75	Lapresa et al.	3	0.05			Simulation filter	Randomization	Figures (with pictures)	
76	Lapresa et al.	3	0.001		Yes		Rand. (Shuf. & rot.)	Tablas	6; 6; 1; 2
77	Lapresa et al.	2	0.05				Randomization (Shuf.)	Tables & figures	
78	Lapresa et al.	2	0.05				Randomization	Tables (with pictures)	7
79	Lavega et al.	4	0.005				Randomization	Figures	
80	Louro et al.							Tables & figures (with diagrams)	
81	Lyon et al.	3	0.0001					Tables & figures	
82	Merlet et al.	3	0.0001					Tables & figures	
83	Pic	4	0.05					Tables & figures	43 (women); 122 (men)
84	Pic	6	0.005					Figures	1402 (girls), 2256 (boys)
85	Pic	3	0.005					Figures	2457 (Premier League); 6228 (La Liga)
86	Pic	3	0.005					Tables & figures	1085 (home teams); 520 (visiting teams)
87	Pic et al.	2	0.05					Figures	
88	Pic et al.	2	0.005					Figures	402 (group 1); 1466 (group 2)
89	Pic et al.	3 and 2	0.005 / 0.05	90%				Tables & figures	In function of parameters
90	Portell et al.	3	0.005			Qual. & quant. filters	Randomization	Figures (with drawings)	2 T-Patterns for each worker
91	Prat et al.							Tables & figures	3
92	Prieto-Lage et al.	5	0.005	90%				Figures	2708 (5 oc); 3238 (3 oc)
93	Prieto-Lage et al.							Figures (with pictures)	
94	Prieto-Lage et al.							Figures (with pictures)	
95	Prieto-Lage et al.							Figures (with pictures)	
96	Prieto-Lage et al.	4	0.05	90%				Tables & figures	14776
97	Prieto-Lage et al.	3	0.005		Yes			Figures	
98	Sandman et al.	3	0.001					Figures	
99	Santangelo et al.		0.0001	80%			Rand. (Shuf. & rot.)	Tables & figures	51
100	Santos et al.	3	0.0005					Figures (with pictures)	2

101	Santos et al.	3	0.001					Tables & figures (with pictures)	16 (Coach 1); 26 (Coach 2); 86 (Coach 3); 105 (Coach 4)
102	Santos et al.	3	0.005	90%		Simulation filter	Randomization	Tables & figures	15 (defensive actions), 17 (offensive actions)
103	Santos et al.	3	0.005					Figures	
104	Santoyo et al.		0.05						133
105	Santoyo et al.	20	0.005	90%	Yes	Qualitative filters	Randomization	Figures	
106	Sastre et al.	3	0.005	90%	Yes		Randomization (Shuf.)	Tables	6
107	Sarmento et al.	3	0.05					Figures	345
108	Sauch et al.							Tables & figures	
109	Sene-Mir et al.	3	0.005			Simulation filter	Randomization		3414 (ses 1), 446 (ses 2)
110	Serna et al.	5	0.005					Figures	37
111	Suárez et al.	7	0.05					Figures	
112	Szekrényes							Tables & figures	
113	Tarragó et al.	2	0.005	90%				Tables	7 (left fencer); 14 (rigth fencer)
114	Tarragó et al.	2	0.005	90%				Tables	15 (male); 18 (female)
115	Tarragó et al.	10	0.005					Tables & figures	72
116	Terroba et al.	2	0.005	90%		Qualitative filters	Randomization (Shuf.)	Tables	
117	Torrents et al.							Figures	
118	Torrents et al.							Figures (with pictures)	663
119	Tripiana	3	0.05					Tables & figures	6
120	Tripiana et al.	3						Tables & figures	3
121	Valero et al.		0.005	90%					
122	Valero et al.	3	0.005					Figures	4
123	Wedl et al.	3	0.001					Tables & figures	
124	Zurloni et al.	7	0.0001					Figures	167
125	Zurloni et al.							Figures	
